# CuFeS_2_ Nanoparticles Functionalized with a Thermoresponsive Polymer for Photothermia and Externally Controlled Drug Delivery

**DOI:** 10.1021/acsami.3c03902

**Published:** 2023-05-03

**Authors:** John S. Conteh, Giulia E. P. Nucci, Tamara Fernandez Cabada, Binh T. Mai, Nisarg Soni, Francesco De Donato, Lea Pasquale, Federico Catalano, Mirko Prato, Liberato Manna, Teresa Pellegrino

**Affiliations:** † Italian Institute of Technology, via Morego 30, 16163 Genoa, Italy; ‡ Dipartimento di Chimica e Chimica Industriale, Università di Genova, Via Dodecaneso 31, 16146 Genoa, Italy

**Keywords:** phototherapy, chalcogenide nanoparticles, chalcopyrite, doxorubicin, chemotherapy, thermoresponsive materials

## Abstract

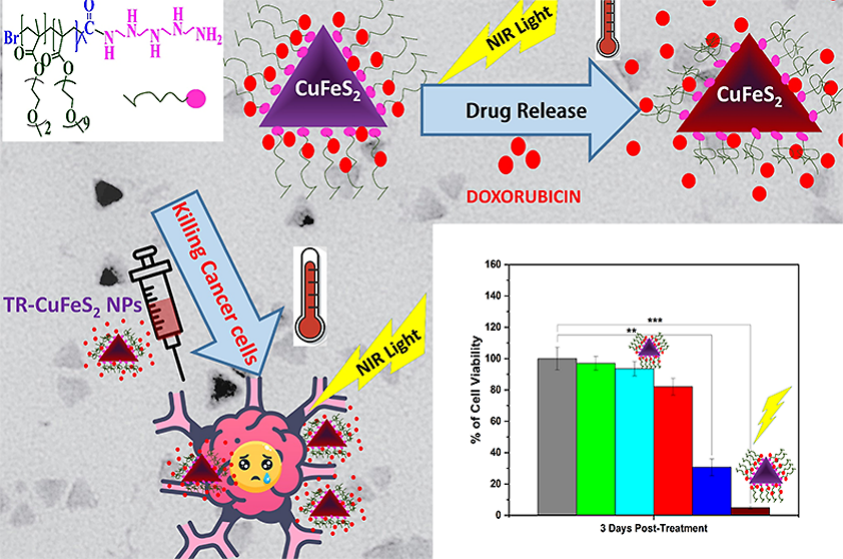

CuFeS_2_ chalcopyrite nanoparticles (NPs) can generate heat under exposure to near-infrared laser irradiation. Here, we develop a protocol to decorate the surface of CuFeS_2_ NPs (13 nm) with a thermoresponsive (TR) polymer based on poly(ethylene glycol methacrylate) to combine heat-mediated drug delivery and photothermal heat damage. The resulting TR-CuFeS_2_ NPs feature a small hydrodynamic size (∼75 nm), along with high colloidal stability and a TR transition temperature of 41 °C in physiological conditions. Remarkably, TR-CuFeS_2_ NPs, when exposed to a laser beam (in the range of 0.5 and 1.5 W/cm^2^) at NP concentrations as low as 40–50 μg Cu/mL, exhibit a high heating performance with a rise in the solution temperature to hyperthermia therapeutic values (42–45 °C). Furthermore, TR-CuFeS_2_ NPs worked as nanocarriers, being able to load an appreciable amount of doxorubicin (90 μg DOXO/mg Cu), a chemotherapeutic agent whose release could then be triggered by exposing the NPs to a laser beam (through which a hyperthermia temperature above 42 °C could be reached). In an *in vitro* study performed on U87 human glioblastoma cells, bare TR-CuFeS_2_ NPs were proven to be nontoxic at a Cu concentration up to 40 μg/mL, while at the same low dose, the drug-loaded TR-CuFeS_2_-DOXO NPs displayed synergistic cytotoxic effects due to the combination of direct heat damage and DOXO chemotherapy, under photo-irradiation by a 808 nm laser (1.2 W/cm^2^). Finally, under a 808 nm laser, the TR-CuFeS_2_ NPs generated a tunable amount of reactive oxygen species depending on the applied power density and NP concentration.

## Introduction

Photothermal therapy (PTT) is a noninvasive and highly selective cancer treatment modality in which a photothermal (PT) agent absorbs light from an external infrared [near-infrared (NIR)] radiation source within the biologically safe wavelength regions (*i.e.*, NIR (I) at 650–850 nm and NIR (II) at 950–1350 nm), and the incident radiation is converted into heat to induce killing of cancer cells.^1−4^ Organic-based dyes such as indocyanine green,^5^ croconaine dye,^6^ porphyrins,^7^ and IR780^8^ have been commonly chosen as PT agents mainly due to their good PT conversion efficiencies, biocompatibility, and facile processability. However, the poor water solubility and rapid photo-degradation of these dye-based PTT agents have limited their applicability in cancer therapy.^9,10^ As an alternative, inorganic nanoparticle (NP)-based systems are generally more stable as PT agents since they can withstand multiple cycles of laser irradiation without undergoing photo-degradation.^9^ This has therefore boosted the synthesis and proof of concept studies to define the potential of various classes of inorganic-NPs-based PT agents.^4,11,12^

In the early developments in this field, gold-based NPs have been very popular as PT agents mainly because they exhibit localized surface plasmon resonance, which is easily tunable by changing their morphology (nanorods,^13^ nanoshells,^14^ nanospheres,^15^ etc.). Although Au NPs are bioinert,^11,12,16−19^ gold-based NPs are not biodegradable *in vivo*, and they tend to accumulate in organs such as the liver and the spleen with poor renal clearance. Therefore, the clinical translation prospects of gold-based NPs remain uncertain.^20−24^ Moreover, most Au-NPs, especially the spherical ones (gold nanospheres), must have quite large hydrodynamic sizes (typically above 100 nm) in order to ensure NIR light absorption.^25^ This large-size attribute might be problematic not only for their cellular internalization but also for renal excretion and NP bioaccumulation.

On the other hand, copper-based chalcogenide NPs, including copper-deficient chalcogenides Cu_2–*x*_E (E = S, Se, Te; *x* = 0–1),^26^ hollow mesoporous copper sulfides,^27^ and chalcopyrite CuFeS_2_^28^ NPs, are now under the spot light as an alternative class of PTT agents.^29−31^ Unlike gold NPs for which the free carrier concentration stays fixed, in copper chalcogenide NPs the free carrier concentration, much lower than that of Au, is tuned by the degree of copper deficiency and is the main parameter that regulates the spectral position of plasmon resonance (which falls in the NIR region of the spectrum) rather than the NP size or shape. In analogy with gold NPs, when plasmon resonance in these NPs is elicited (in this case, by absorption of NIR radiation) the photoexcited carriers (in this case, valence band holes) then relax by releasing heat, hence generating the observed PT effect.^12,29,31^ Since copper chalcogenide NPs of a few nm size exhibit NIR plasmon resonance, they are seen as more advantageous over Au NPs for cancer therapy. This is because by being so small, yet excitable by NIR light, they can more easily accumulate in tumors by means of the enhanced permeability and retention effect.^32,33^ Also, under physiological-like conditions, copper chalcogenides having sulfur atoms in their structure were proven to biodegrade, forming water-soluble copper sulfates,^34^ and *in vivo* studies using mouse models have also demonstrated the biodegradability or clearance of CuS and Cu_2–*x*_Se NPs in a nontoxic manner (the slow and controlled release of copper can result in a controllable NP toxicity).^35,36^

In comparison to gold NPs, copper-based NPs are cheaper to produce, less toxic, and have much higher PT conversion efficiencies.^21,34,37−39^ Besides their use as PTT agents, they can as well serve as contrast agents in photoacoustic and PT imaging.^29,40^ Finally, upon interaction with light in aqueous medium and in the presence of molecular oxygen, copper chalcogenides can generate appreciable amounts of radical oxygen species (ROS), *e.g.*, hydroxyl radicals (^•^OH), singlet oxygen (^1^O_2_), and peroxides (ROO^•^), which are used to induce lipid peroxidation, DNA injury, protein damage, and ultimately cell death in the photodynamic therapy of cancers.^37,41,42^ As a typical member in this group of copper-based NPs, chalcopyrite (CuFeS_2_) NPs are n-type semiconductors with an unusually low optical band gap of 0.53 eV. This material absorbs remarkably in the NIR biological window and exhibits outstanding PT efficiencies (49–82%) in aqueous media.^43−45^ A way to enhance the therapeutic efficacy of PTT is to combine it with other therapies, such as magnetic hyperthermia,^46^ radiotherapy (RT),^47^ and chemotherapy (CT).^13^ Among them, the combination of PTT and CT is the most straightforward strategy to provide synergic anticancer effects that are more effective than the individual therapies.^13,48,49^ To this purpose, in some works, inorganic NP-based PT and CT agents were co-encapsulated in a responsive block copolymer. A typical example of such a system was reported by Liao *et al.* who encapsulated gold nanorods and doxorubicin (DOXO) in a block copolymer matrix, polyethylene glycol-b-polycaprolactone (PEG-b-PCL), which forms a polymersome.^13^ In another approach, the inorganic-NP PT agents were functionalized with a polymer moiety, displaying either a pH, enzymatic, ROS, or thermoresponsive (TR) behavior.^20,26,50−53^ For instance, Huang’s group synthesized a system comprising copper sulfide NPs functionalized with the TR-polymer, poly(*N*-isopropylacrylamide-*co*-methyl methacrylate) (Cu_1.75_S@p(NIPAM-MAA)).^33^ The DOXO drug was then loaded into these large-size NPs (*ca.* 153 nm) and, upon irradiation with a 808 nm laser, the NPs solution was heated at a temperature above the polymer’s lower critical solution temperature (>LCST), causing the shrinkage of the polymer and the release of 80% of the loaded drug. In a similar effort, Ortiz de Solorzano’s group synthesized hollow CuS NPs decorated with a biodegradable copolymer made of oligo(ethylene glycol) methyl ether methacrylate, namely poly(DEGMEMA-*co*-OEGMEMA, with a size typically greater than 100 nm, and then they encapsulated the bupivacaine drug in these NPs.^32^ To date, most of the copper-based NPs and TR-based nanocarriers have exploited monometallic chalcogenide NPs that were generally found to be in clusters of multiple inorganic cores and had rather high hydrodynamic sizes, typically above 100 nm, with tumor/intracellular delivery and degradation challenges to face.

In this context, CuFeS_2_ NPs could be much more suitable for such heat-triggered delivery of CT agents, given their outstanding PT properties mentioned earlier, including their high molar extinction coefficient (ε = 5.2 × 106 M^–1^ cm^–1^) in the NIR region (specifically 808 nm).^28,43,54^ So far, the biomedical applications of chalcopyrite NPs have mainly been restricted to their use as standalone PTT agents or in multimodal platforms (namely combining chalcopyrite NPs with photoacoustic imaging, magnetic resonance imaging, or computed tomography imaging).^44,45^ In a very first attempt, Chang and co-workers reported the use of chalcopyrite NPs in combined chemo-PTT applications. In their work, the CuFeS_2_ NPs co-encapsulated with cisplatin-functionalized hyaluronic acid displayed an acidic pH and glutathione-enzyme-responsive behavior. The cisplatin-NPs were proven *in vitro* on B16F1 and HeLa cancer cells to be efficient in pH-mediated drug release (CT) in synergy with photo-mediated heat damage.^28^

We report here the development of a system based on TR-polymer-functionalized TR-CuFeS_2_ NPs for use in externally triggered drug delivery and PTT cancer treatment. By means of a “grafting to” approach, the surface of pristine CuFeS_2_ NPs (13 ± 1 nm in size with a pyramid shape) was coated with thermoresponsive poly(oligo ethylene glycol)methyl methacrylate-*co*-diethylene glycol methyl ether methacrylate. This is a biocompatible polymer, prepared by photo-induced atom-transfer radical polymerization (photo-ATRP).^55^ The resulting TR-CuFeS_2_ featured a small hydrodynamic size and optimal colloidal stability in physiological conditions, along with the preservation of their photo-heating performance. Moreover, we found that significant amounts of ROS were generated from the TR-CuFeS_2_ solution under laser exposure, and these were strongly dependent on the laser power density and NP concentration. Finally, the TR-CuFeS_2_ NPs at Cu concentration as low as 40 μg/mL were found to be biocompatible *in vitro*, and they could load an appreciable amount of DOXO drug (90 μg DOXO/mg Cu). In turn, DOXO could be released upon exposure to laser and only when reaching a therapeutic temperature of 45 °C; the *in vitro* cytotoxicity study on the U87 malignant glioma cell model demonstrated the synergistic effect between PTT and on-demand CT release of our TR-CuFeS_2_ NPs.

## Results and Discussion

### Synthesis and Characterization of TR-CuFeS_2_ NPs

The 13 ± 1 nm (edge size) CuFeS_2_ NPs used in our study were prepared by colloidal hot-injection synthesis, as reported in a previous work.^43^ That work also showed that these small-size NPs, once transferred in water with a mercapto polyethylene glycol polymer, upon 808 nm laser irradiation, exhibited a high PT conversion efficiency of 49%, hence representing interesting PT candidates.^43^ Here, to further obtain TR-polymer-functionalized CuFeS_2_ NPs, we grafted to the NP surface a tetra-amine terminated TR-polymer. This was achieved using a simple and straightforward ligand exchange procedure with a presynthesized TR-polymer, according to the preparation protocol described below.

#### Synthesis of TR-Polymer

**Figure 1:**
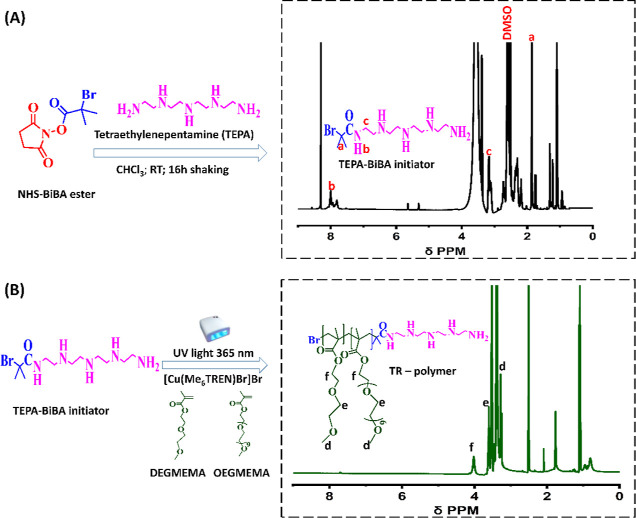
Reaction schemes of (A) ATRP initiator synthesis *via* aminolysis reaction carried out in chloroform at room temperatures and (B) TR-polymer synthesis using photo-ATRP with their corresponding ^1^H NMR spectra, respectively.

The TR-polymer was synthesized by the photo-ATRP technique following a two-step synthesis (Figure 1). Photo-ATRP enables better control of the polymerization while using less amount of copper(II) catalyst (only few ppm) than classical ATRP. At the same time, it reduces the cost and time required for catalyst removal after polymerization.^55−57^ In the first step of the TR-polymer synthesis, a bifunctional ATRP initiator (TEPA-BiBA) having a tetra-amine and pseudo-halide group was synthesized by an aminolysis reaction between *N*-hydroxysuccinimide bromoisobutanoate ester (NHS-BiBA) and tetraethylenepentamine (TEPA) (Figure 1A). The bromo halide end of this customized initiator served as the starting point to promote polymer chain growth, while the tetra-amine moieties were later exploited to anchor the TR-polymer to the surface of CuFeS_2_ NPs. The chemical structure of TEPA-BiBA after its synthesis was confirmed by means of ^1^H NMR (Figure 1A): the singlet peak at 1.8 ppm (a) is attributed to the two methyl groups (BrC(**CH**_**3**_)_2_CO), while that at 8.0 ppm (b) represents the proton of the amide group (C(=O)**NH**). The triplet peak at 3.2 ppm is assigned to the methylene protons (CONH**CH**_**2**_CH_2_) closer to the amide bond.

In the second step, the goldish-yellow TEPA-BiBA was then used as an initiator to synthesize the TR-polymer containing diethylene glycol methyl ether methacrylate [DEGMEMA] and hydrophilic oligoethylene glycol methyl ether methacrylate [OEGMEMA_500_] monomer units (Figure 1B). In this photo-ATRP polymerization carried out in dimethyl sulfoxide, a mixture containing copper bromide (CuBr_2_) and tris[2-(dimethylamino)ethyl]amine (Me_6_TREN) was used to grow the TR-polymer. Here, the ratio of [monomer]/[initiator]/[Me_6_TREN]/[CuBr_2_] was set to 20:1:0.08:0.04, respectively. To avoid overheating of the polymerization vessel upon exposure to UV light, which normally hampers polymerization control, the reaction vessel was placed in a cold room set at 5 °C. The resulting crude P(DEGMEMA-*co*-OEGMEMA_500_) polymer obtained after 6 h of polymerization was first filtered through an aluminum oxide packed column to remove the copper catalyst, followed by three cycles of diethyl ether precipitation, centrifugation, and tetrahydrofuran washes. Despite following this thorough cleaning procedure, the obtained TR-polymer was noticeably pale blue in color, indicating the residual presence of the Cu catalyst likely chelated to the tetra-amine terminal of the TEPA-BiBA initiator. Indeed, inductively coupled plasma (ICP) analysis of the cleaned polymer confirmed the retention of about 0.16 mg of copper catalyst per 13 mg of washed TR-polymer. However, since the TR-polymer is used here for Cu-based NPs, this issue does not hamper the ligand exchange process, as shown in the next section.

Proton NMR of the resulting TR-polymer (Figure 1B) indicates the presence of a singlet peak at 3.2 ppm (d) which can be attributed to the methoxy protons (−O(CH_3_)), while the peak at 3.5–3.6 ppm (e) represents the ethylene group of the polymer side chains. The singlet at 4.0 ppm (f) can be assigned to the protons of the methylene group closest to the carbonyl functionality. Noticeable in this TR-polymer spectrum (Figure 1B) is the absence of the resonance of the proton from the amide group that was initially present in the TEPA-BiBA initiator (at δ 8.0 ppm) (Figure 1A, black spectrum). This lack of signal may be explained by the chelation of the amide groups to the residual copper catalyst amount, as mentioned above.

With the aim to achieve a polymer composition having a phase-transition temperature (the LCST) above 37 °C, the molar percentage ratio of the amphiphilic (DEGMEMA) to the hydrophilic (OEGMEMA) monomer was set to 88:12. To roughly evaluate the polymer’s LCST, we used a water bath. We first performed a quick check of ‘cloud point’ of the TR-polymer in the range from 25 to 50 °C, with 5 °C steps, to identify the temperature at which the clear TR-polymer solution became cloudy due to the lack of polymer hydrophilicity at temperatures above LCST. Using this temperature of the ‘cloud point’ as reference, the precise polymer LCST was determined by dynamic light scattering (DLS) measurements.^58^ Here, the change in hydrodynamic size (*D*_h_) of the polymer in solution measured by DLS against temperature was observed at 42 °C, and it corresponded to the LCST of our synthesized TR-polymer (Figure S1A). We also determined the average molecular weight of the TR-polymer by a static light scattering method.^59^ This is done by plotting scattering intensity *versus* concentration: from the reciprocal of the Rayleigh and Debye plot’s intercept, it is possible to estimate the TR-polymer’s average molecular weight, which was 4300 g/mol in our case (Figure S1B).

#### Ligand Exchange Polymer Procedure

**Figure 2:**
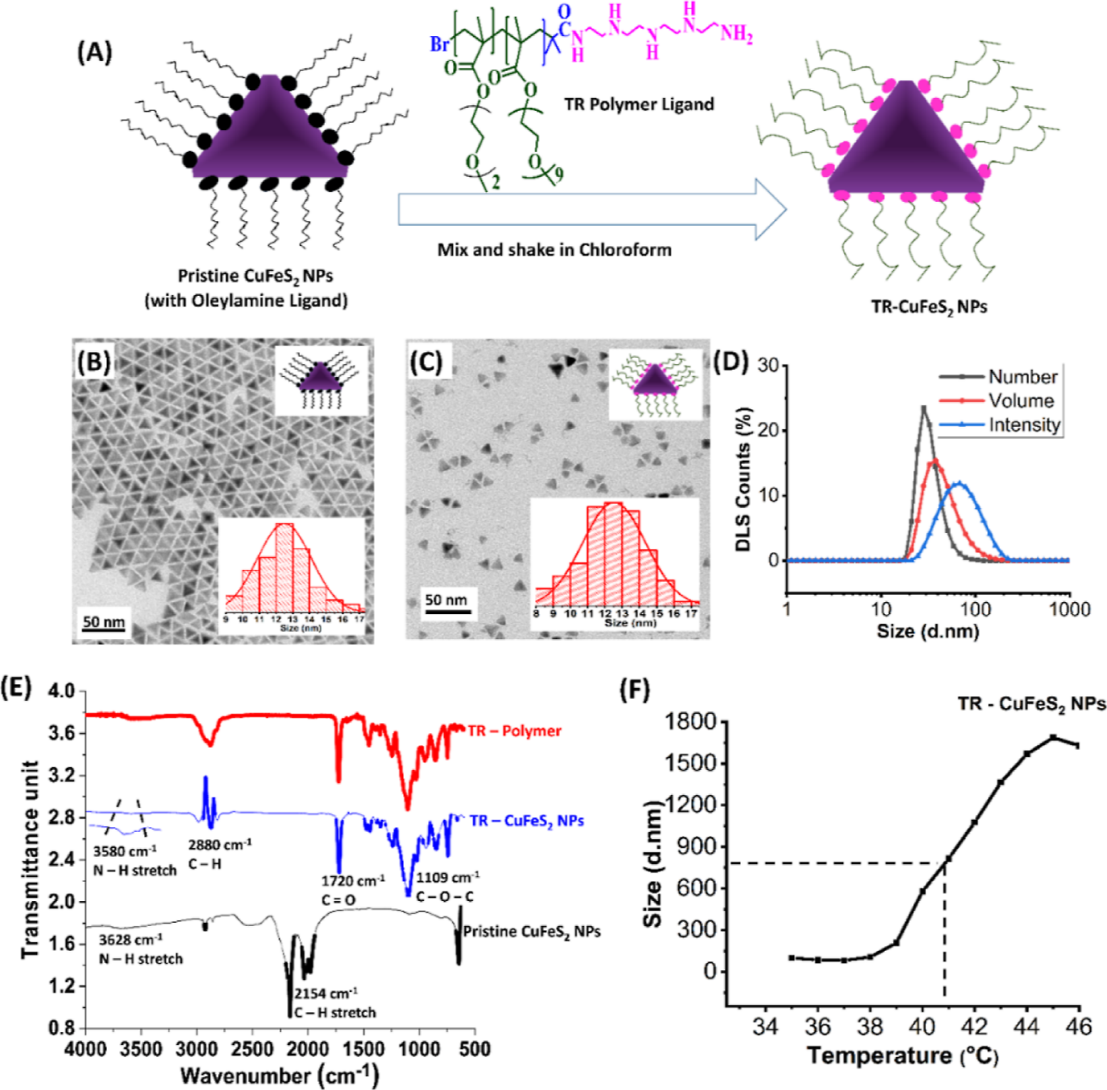
Synthesis and characterization of TR-CuFeS_2_ NPs in water. (A) Ligand exchange reaction Scheme, (B,C) TEM image with corresponding size histograms of pristine CuFeS_2_ NPs in chloroform and TR-CuFeS_2_ NPs (50 ligand/nm^2^) dispersed in water, respectively (D) DLS hydrodynamic sizes (*D*_h_) weighted by number, volume, and intensity (PDI = 0.21) for TR-CuFeS_2_ NPs obtained at the optimized experimental conditions (ligand/nm^2^ ratio of 50, ligand exchange time = 48 h), (E) FT-IR spectra of the pristine CuFeS_2_ NPs (black), TR-CuFeS_2_ NPs (blue), and TR-polymer (red), confirming ligand exchange success and (F) LCST of TR-CuFeS_2_ NPs done by DLS measurement of change in NP’s hydrodynamic size with temperature.

The tetra-amine-terminated TR-polymer was successfully grafted to the surface of the pristine pyramid-shaped CuFeS_2_ NPs by means of a ligand exchange protocol (Figure 2A). Indeed, thanks to the high affinity of the amine functionalities toward the Cu-rich surface of CuFeS_2_ NPs, the exchange reaction was carried out by simply mixing the TR-polymer and the pristine CuFeS_2_ NPs in chloroform at room temperature, under gentle shaking. For this exchange, the polymer ligand number per squared nanometer (nm^2^) of NP surface and the reaction time were among the most important parameters to set in order to obtain colloidally stable TR-CuFeS_2_ NPs. The best protocol was optimized at a ligand/nm^2^ ratio of 50, and the best reaction time was found to be 48 h. Using optimal conditions, single-coated water-dispersed TR-CuFeS_2_ NPs were obtained, as confirmed by the absence of aggregates on the transmission electron microscopy (TEM) grids of a water-soluble sample. Also, the size (*ca.*13 nm) and the pyramid shape of the pristine CuFeS_2_ NPs were well-preserved (Figure 2B,C). In agreement with TEM, DLS traces also revealed the aqueous hydrodynamic size of the TR-CuFeS_2_ NPs weighted by number, volume, and intensity with a monomodal profile, and the peaks centered at 32 ± 10, 46 ± 22, and 75 ± 34 nm (polydispersity index, PDI = 0.21), an indication of single-coated and well-dispersed colloidal NPs (Figure 2D).

For ligand/nm^2^ ratios below 50, the resulting TR-CuFeS_2_ NPs were very difficult to disperse in water and TEM images revealed that they were aggregated (Figure S2). Instead, at a ligand/nm^2^ ratio of 50, the exchange times of 48 and 72 h led to water-soluble NPs without any notable differences in terms of structure and size, and therefore the shortest time was chosen (data not shown).

To further verify the success of the ligand exchange procedure, FT-IR characterization was performed on the resulting TR-CuFeS_2_ NPs and compared to the pristine CuFeS_2_ NPs and to the TR-polymer ligand used for the surface functionalization (Figure 2E). Starting from the pristine CuFeS_2_ spectrum (black curve), the band at 2154 cm^–1^ is attributed to the C–H stretching of the oleylamine ligand initially present on the surface of the NPs. This disappeared on ligand exchange in the TR-CuFeS_2_ NPs spectrum (blue curve). Most importantly, the sharp peaks at 1720 cm^–1^ (the stretching band of C=O groups) and 1110 cm^–1^ (C–O–C stretching vibrations) indicate the presence of TR-polymer components. The FT-IR spectrum of TR-CuFeS_2_ NPs resembles that of TR-polymer in the region below 1750 cm^–1^ (Figure 2E, red curve). On the other hand, the peak at 3580 cm^–1^ is ascribed to N–H stretching and that at 2880 cm^–1^ is assigned to the C–H vibrational stretching of PEG (Figure 2E, blue curve).

X-ray photoelectron spectroscopy (XPS) was employed to monitor the changes in surface composition after each functionalization step (polymer coating and drug loading). The wide-scan spectrum collected for the pristine CuFeS_2_ NPs (Figure S3A, blue plot) showed the expected signals for the inorganic core (Cu, Fe, and S) and the signals from the organic shell (C and N), together with traces of surface oxidation/environmental contamination (O signal). On the TR-CuFeS_2_ NPs, the intensity of the signals related to the inorganic core of the NPs now embedded with the TR-polymer drastically dropped (Figure S3A, green plot), while the C and O signals intensity increased, likely due to the polyethylene oxide chains of the TR-polymer coating. The presence of bromine (Br) associated with the TR-polymer in the high-resolution XPS spectra (Figure S3B(iii)) further supported the TR coating. The loading of DOXO for the TR-CuFeS_2_-DOXO NPs instead further decreased the Cu, Fe, and S core’s signal intensities, and at the same time, the C and O signal intensities further increased (Figure S3A, orange plot). Taking into consideration the surface sensitivity of XPS and its probing depth (10 nm), these observations suggested the successful TR-functionalization of the NPs.

Next, we determined the LCST of the TR-CuFeS_2_ NPs using the DLS technique discussed in the previous section for the TR-polymer. The *D*_H_*versus* temperature curve shown in Figure 2F indicates an inflection point at 41 °C for the TR-CuFeS_2_ NPs, and this value is slightly lower than the LCST obtained for the TR-polymer (42 °C). However, an LCST of 41.0 °C is still higher than the body temperature and within the range of PT therapeutic temperature, thus being suitable for our heat-triggered drug delivery applications.^53^

### PT Characterization of TR-CuFeS_2_ NPs

**Figure 3:**
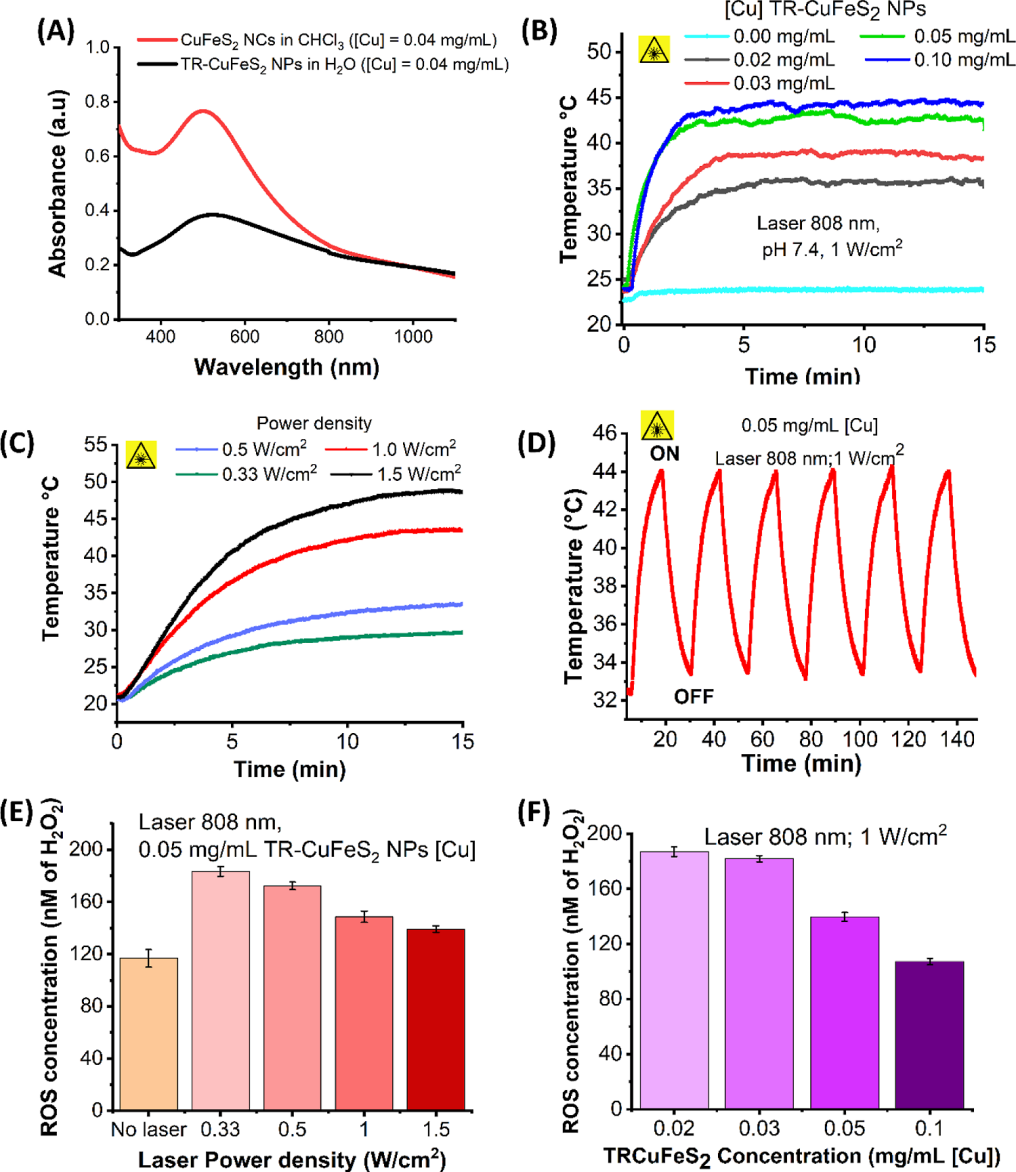
PT and ROS release characterization of TR-CuFeS_2_ NPs. (A) UV–vis spectra of pristine CuFeS_2_ NPs in chloroform (red curve) and TR-CuFeS_2_ NPs in water (black curve) measured at 0.04 mg_[Cu]_/mL. Although the overall spectra are quite modified, the two samples show similar absorption at a wavelength of 808 nm. At this same wavelength, the molar extinction coefficient (ε) of TR-CuFeS_2_ NPs in water was determined to be 1.82 × 10^7^ M^–1^ cm^–1^; (B) heating profiles of NPs at different doses (Cu concentrations) under a 808 nm laser at (1.0 W/cm^2^); (C) heating profiles obtained by varying the power density of a 808 nm laser for NPs concentrations of 0.05 mg/mL [Cu]); (D) heating profile of multiple cycles of 808 nm-photo-irradiation (1.0 W/cm^2^) of TR-CuFeS_2_ NPs (0.05 mg/mL [Cu]), and (E,F) Effect of laser power density and NP dose on the concentration of ROS generated by TR-CuFeS_2_ NPs upon laser irradiation, respectively. (The quantified NPs ROS are expressed in terms of hydrogen peroxide concentrations [H_2_O_2_].

Prior to characterizing the PT properties of our TR-CuFeS_2_ NPs, we first compared the optical spectra of the pristine CuFeS_2_ NPs (in chloroform) to that of TR-CuFeS_2_ NPs in water, at similar copper concentration of 0.04 mg/mL, to establish whether the TR-polymer coating affects the spectral properties of the CuFeS_2_ core. Although we recorded some significant changes in the UV–vis absorbance spectra in the aqueous media, a significant absorption at 808 nm was still present for the CuFeS_2_ NPs, which is the region of interest for our PTT (Figure 3A). At this 808 nm wavelength, the molar extinction coefficient of CuFeS_2_ NPs in water was calculated to be 1.82 × 10^7^ M^–1^ cm^–1^ (see Figure S4, Table S1, and the protocol for molar extinction coefficient determination in the Supporting Information for more details).

Next, to evaluate the efficiency at which NIR light absorbed by TR-CuFeS_2_ NPs is converted into heat, the PT conversion effect of TR-CuFeS_2_ NPs was measured by adopting a protocol reported elsewhere in the literature and here detailed in the Supporting Information.^43,60^ The PT conversion efficiency of TR-CuFeS_2_ NPs was determined to be 47.8% (Figure S5 and Table S2). This value does not differ much from the 49% reported earlier for the same CuFeS_2_ NPs but coated by a mercapto-PEGylated ligand.^43^

Having ascertained the good light to heat conversion property of TR-CuFeS_2_ NPs using a laser at 808 nm operating at a constant power density of 1.0 W/cm^2^, we investigated the change in temperature of a TR-CuFeS_2_ NPs saline solution as a function of the NPs dose in terms of Cu concentrations (Figure 3B). In comparison to a pure saline solution (purple curve, Figure 3B), the TR-CuFeS_2_ NPs at all concentrations tested, in the range 0.02–0.1 mg/mL [Cu], produced a significant rise in temperature within few minutes of excitation. For instance, at a copper concentration of 0.1 mg/mL, the NPs heated up to ∼45 °C (the therapeutic temperature) from room temperature, making a net temperature gain of *ca.* 22 °C.

The temperature dependence of the TR-CuFeS_2_ NPs on the power density at 808 nm was also evaluated by irradiating a saline solution of NPs (0.05 mg/mL [Cu]) for 15 min at different power densities, ranging from 0.33 to 1.5 W/cm^2^. A visible maximum temperature reached with power density was recorded (Figure 3C). For instance, at power densities of 0.33, 1.0, and 1.5 W/cm^2^, we obtained maximum temperatures of ∼30, ∼43, and ∼48 °C with respect to the starting solution temperature of 20 °C, corresponding to a temperature difference of 10, 23, and 28 °C, respectively. Interestingly, even under mild irradiation conditions (0.5 W/cm^2^, 0.05 mg/mL [Cu]), the TR-CuFeS_2_ NPs displayed a significant temperature gain of ∼ 12 °C, a temperature difference that is enough to ensure reaching the therapeutic temperature range (41–45 °C), given the body temperature of 37 °C (Figure 3C, blue curve). Interestingly, upon multiple cycles of laser exposure of 15 min each, the TR-CuFeS_2_ NPs did not lose their heating performance (consistently reaching a final temperature of ∼44 °C), and the material remained noticeably stable after these robust heating and cooling cycles (Figure 3D). This demonstrates the potential to apply multiple PTT cycles as often done in *in vivo* studies.

### ROS Release Characterization

In an aqueous medium and in the presence of molecular oxygen, chalcogenide NPs can generate ROS species (*e.g.*, ^•^OH, ^•^O^2–^, ^1^O_2_, etc.) upon laser irradiation (Figure 3D), which may cause oxidative stress and cell death in cancer cells.^2,61,62^ To quantify the ROS generated by TR-CuFeS_2_ NPs, a class of chalcogenide NPs that has not yet been studied, we used a fluorescence assay based on dichlorofluorescein diacetate (DCFH-DA) dye which upon its reaction with ROS undergoes deacetylation to form highly fluorescent 2,7-dichlorofluorescein (DCF) dye.^9,63^

Here, we first investigated the dependence of ROS generated by TR-CuFeS_2_ NPs on the power density variation. For this, saline solutions of TR-CuFeS_2_ (0.05 mg/mL Cu concentration) were irradiated for 15 min at various power densities (0.33 to 1.5 W/cm^2^). As control experiments, two samples not exposed to irradiation were prepared. One of these samples consisted of just the saline medium and the other was the NP solution (0.05 mg/mL Cu) with both being mixed with the DCFH-DA dye. Both the irradiated and non-irradiated samples were incubated for 2.5 h, and the resulting fluorescence of the DCF dye was monitored (Figure S6A) on the supernatant of their respective solutions after having removed the NPs by centrifugal filtration. To actually quantify the total amount of ROS generated from each experimental condition, a calibration plot (Figure S6C, see ROS quantification section in the Supporting Information for more details on curve preparation) was prepared using hydrogen peroxide (H_2_O_2_) as a model ROS species due to its stability and long life span as opposed to the other forms of ROS species.

As evidenced in Figure 3E, the generation of ROS species by our NPs during irradiation was confirmed by the significant boost in ROS amounts starting from the basal concentrations in the control samples. The study also surprisingly revealed that higher power densities (thus higher temperatures) led to a decreasing amount of generated ROS (Figure 3E). For instance, upon irradiating the NP solution using 0.33 W/cm^2^ power density, the nominal concentration of ROS generated was 1.84 ± 0.10 (nM H_2_O_2_), while at a much higher power density (1.5 W/cm^2^), the ROS concentration decreased to 1.39 ± 0.07 (nM H_2_O_2_). To evaluate the amount of ROS generated as a function of the NP concentration, NPs samples at [Cu] ranging from 0.02 to 0.1 mg/mL were irradiated at a constant power density of 1.0 W/cm^2^ while maintaining a pH of 7.4 for all experiments (Figure 3F). The study revealed that lower amounts of ROS were produced at higher NP concentrations. For example, at a concentration of 0.02 mg/mL [Cu], the NPs generated a ROS concentration of about 1.82 (nM H_2_O_2_), a value much higher than the 1.04 (nM H_2_O_2_) estimated for a NP solution with copper concentration of 0.1 mg/mL. Remarkably, this observation as well as the trend observed in the ROS dependence on the power density is opposite to the one reported for Cu_2–*x*_S NPs^64^ and gold NPs.^65^ As such, we hypothesized the possible leaching of Fe^2+^ species from the TR-CuFeS_2_ NP’s core, in a temperature-dependent manner, which might be consuming part of the generated ROS by means of a Fenton-type reaction.^66^

To support this claim, quantification of Cu and Fe element leakage by elemental analysis [ICP–optical emission spectroscopy (OES)] was performed to compare the same sample of TR-CuFeS_2_ NPs (0.5 mgFe/mL) in the presence (15 min laser 808 irradiation) or the absence of laser treatment. The elemental analysis (Cu and Fe) conducted on the NP residues and on the solution filtrate upon centrifugal separation suggests an appreciable amount of Fe (0.24 ± 0.01 mg/mL) in the filtrate portion of the laser-exposed sample, with no such leakage observed in the filtrate of the sample without laser exposure (Table 1). This Fe leakage from the TR-CuFeS_2_ NP’s core into the solution during irradiation was further justified by the decrease in the Fe to Cu ratio observed in the residue of the laser-exposed sample (0.77) in comparison with that without laser treatment (0.82). This iron leakage may justify the lowering of the overall amount of ROS found in solution after irradiation. Interestingly, we also observed that this Fe leakage does not adversely affect the NPs’ morphology, as revealed by TEM characterization (see Figure S7), or their heating performance over this period. It is also important to mention that thermal stability under irradiation of the fluorescent DCF dye at pH 7.4 was also tested by monitoring the photo luminescent signal of the dye at different temperatures, and no signal change was observed. This experiment ruled out thermal degradation of the dye during laser irradiation.^9^

**Table 1:** Comparative ICP–OES Quantification Study of TR-CuFeS_2_ NPs Elemental Cu and Fe Compositions with or without Laser 808 Irradiation

exp	sample	Cu (mg/mL)	Fe (mg/mL)	Fe/Cu
1	residue of TR-CuFeS_2_ NPs (no laser)	3.13 ± 0.01	2.56 ± 0.03	0.82
2	filtrate of TR-CuFeS_2_ NPs (no laser)	0.00	0.00	
3	residue of TR-CuFeS_2_ NPs (with laser)	3.79 ± 0.02	2.93 ± 0.02	0.77
4	filtrate of TR-CuFeS_2_ NPs (with laser)	0.00	0.24 ± 0.01	

#### Drug Loading and Release Study

**Figure 4:**
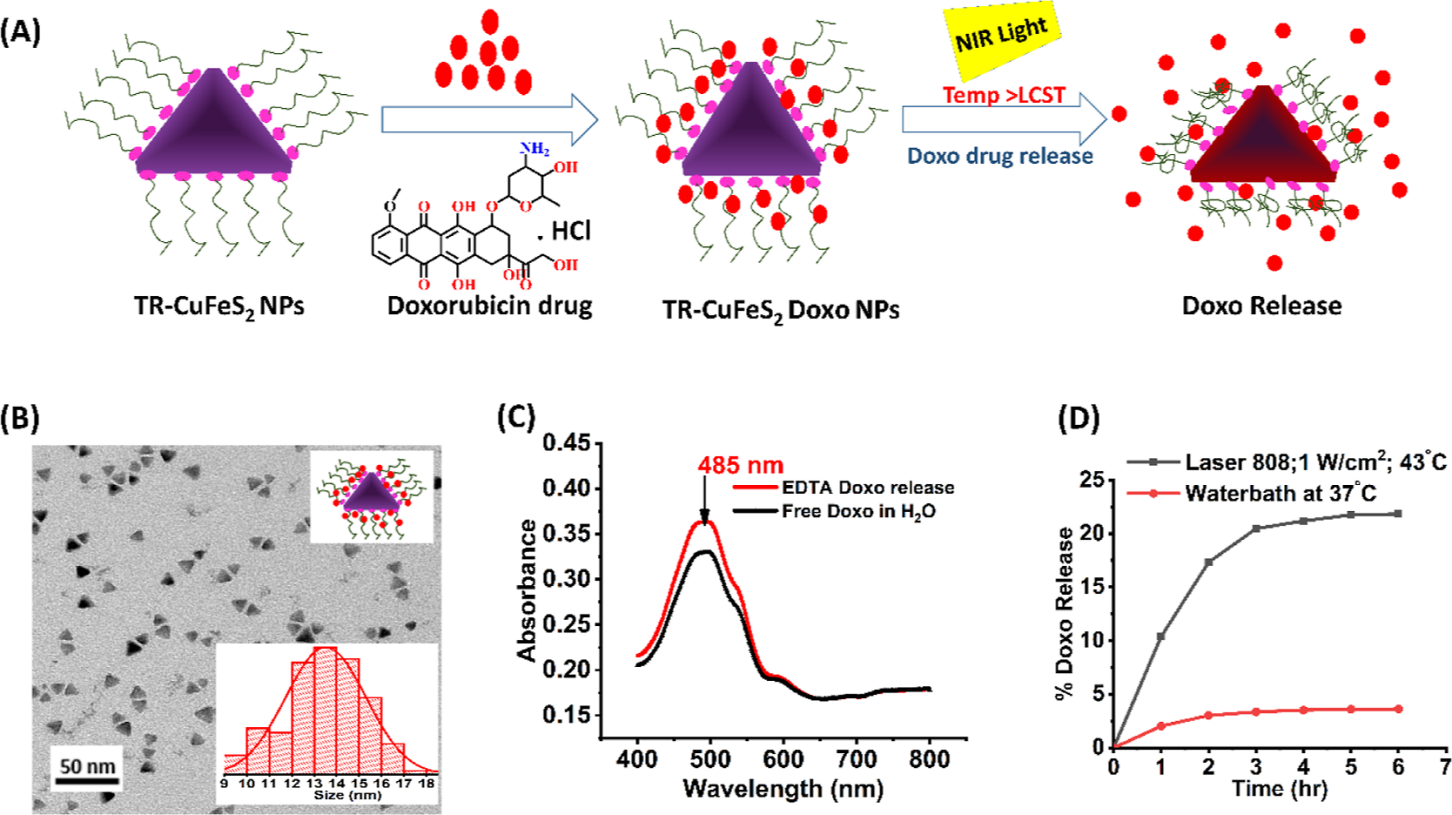
DOXO loading and release characterization of TR-CuFeS_2_-DOXO NPs. (A) Reaction scheme of DOXO drug loading and release; (B) TEM image with histogram size inset of TR-CuFeS_2_-DOXO NPs; (C) absorption spectra of free DOXO in water and the DOXO release from TR-CuFeS_2_-DOXO NPs in the presence of ethylenediaminetetraacetic acid (EDTA); and (D) comparative cumulative DOXO release for TR-CuFeS_2_-DOXO NPs (0.5 mL and 0.1 mg/mL [Cu], pH 7.4) in a water bath at 37 °C (red plot) and an 808 under laser at 43 °C (1.0 W/cm^2^) (black plot).

We then moved a step forward to study the loading and release of DOXO, as an anticancer drug within the TR-polymer shell (Figure 4A).^67^ The loading of DOXO onto TR-CuFeS_2_ NPs was achieved by simply mixing the NPs (0.02 mg/mL Cu) with free DOXO (0.01 mg/mL) in Milli-Q water over an intended period at RT, and the excess of unloaded drug was washed off by centrifugal filtration. Here, the loading was affected by the interaction between DOXO/TR-CuFeS_2_, which is mostly due to the hydrogen bonds formation between DOXO and the grafted TR-polymer and, also, given the slightly negative surface charge (−6.90 mV) of TR-CuFeS_2_ NPs as measured by zeta potential measurements (Figure S8A) and positive charge of the hydrochloric acid-functionalized DOXO, electrostatic attraction between the drug and NPs could not be excluded. Finally, as previously reported,^68^ it cannot be excluded that the DOXO drug forms complexes with Fe or Cu ions at the NP surface, facilitating the drug loading.

At pH 7.4, the NPs and DOXO were colloidally stable in the solution for quite a long time. Only after 14 h, the TR-CuFeS_2_ NPs precipitated out of the solution leading to aggregates, as evidenced by TEM characterization (Figure S8B). This effect may occur because of the oversaturated loading of the DOXO drug on the NPs. Using the optimal drug-loading conditions (pH of 7.4 and loading time of 6 h), we obtained well-dispersed TR-CuFeS_2_-DOXO NPs in water, as confirmed by the TEM image with inset size distributions (Figure 4B), which do not show any significant size difference with respect to the TR-CuFeS_2_ NPs. Similarly, the hydrodynamic size of the DOXO-loaded TR-CuFeS_2_ NPs (weighted by number, volume, and intensity) point to no aggregation (same mono-modal *D*_H_ peaks weighted by number, volume, and intensity) as evidenced in Figure S8C. The same optimized loading conditions (NPs and DOXO concentration, reaction time) could be applied to the loading in saline solution.

Worthy of note is that earlier efforts to perform the loading in Milli-Q water/saline solution at pH 5.0 (a pH value more suitable to promote DOXO solubility) proved unsuccessful, as the NPs were noticeably unstable and precipitated out of the medium immediately after addition. Also, the loading of DOXO in PBS medium was unsuccessful as in this case DOXO was precipitating out of the solution (data not shown).

In view of future *in vivo* application, acquiring some data about saline stability of the NPs is relevant: the hydrodynamic sizes of both TR-CuFeS_2_ and TR-CuFeS_2_-DOXO NPs were evaluated over an 8 day period in 0.9% saline solution using DLS. From the comparison of the traces of the DLS intensity peaks, no significant variation of their monomodal peaks over time was recorded (Figure S9A,B). In addition, by a visual inspection of the samples in saline solution, no visible changes were captured between the sample solutions at day 0 and day 8 (see insets in Figure S9A,B). Overall, these data suggest that the NPs were stable in saline media.

#### DOXO Quantification

We adopted a recently reported protocol to determine the total amount of DOXO loaded on our TR-CuFeS_2_-DOXO NPs.^69,70^ In brief, to the NPs dispersed in Milli-Q water an EDTA solution was added, and the mixture was acidified by dilute HCl addition. Next, it was heated up to 60 °C to ensure complete release of the encapsulated drug following shrinkage of the surface-grafted TR-polymer shell. Here, EDTA was required as a copper and iron chelator ligand to prevent the possible interaction of the Cu ions of the NPs’ core with the released DOXO, forming an undesired Cu/Fe–DOXO complex that could interfere with the UV–visible signal spectra of DOXO or even decrease the quantifiable amount of the drug.^69^ After centrifugal separation of the TR-CuFeS_2_-DOXO NPs, the supernatant containing the released DOXO was quantified by UV spectrometry with the drug’s peak appearing at the 485 nm wavelength mark (Figure 4C, red curve) and showing a profile comparable to that of free DOXO in water (Figure 4C, black curve). At the optimized loading conditions, we calculated an efficiency of DOXO encapsulation of 29.2 ± 1.9%, while the drug-loading weight amount corresponded to 90 μg DOXO per mg of Cu in the NPs dose (Table S3). This loading efficiency is slightly lower than that achieved by Huang *et al.* for the NIPAM-functionalized CuS NPs (40% after 72 h of loading)^33^ but higher than that reported by Li, *et al.* using PEGylated hollow CuS NPs (10% within 5 days).^69^ Apparently, our increase in the loaded amount of DOXO might be due to the higher surface-to-volume ratio of our small-sized TR-CuFeS_2_ NPs compared to their hollow CuS NPs.^71^

#### In Test Tube DOXO Release Study

In a test tube, we first evaluated the DOXO amount release, laser-induced, by irradiating the TR-CuFeS_2_-DOXO NPs sample (saline, pH 7.4, 37 °C starting temperature) with a 808 nm laser operated at 1.0 W/cm^2^ and reaching a temperature of 43 °C, monitoring the DOXO release every hour with the EDTA release protocol described above. In a similar manner, we evaluated the amount of nonspecific drug release occurring at physiological conditions by immerging the saline solution of TR-CuFeS_2_-DOXO NPs in a water bath kept at 37 °C. The release experiments were conducted for up to 6 h, monitoring the same batch of NP samples and evaluating the cumulative DOXO release profiles (Figure 4D). As expected, the use of laser induced a higher amount of DOXO release in comparison to that of the sample kept at 37 °C. Here, TR-CuFeS_2_-DOXO released about 25% of the loaded drug over a 6 h period (0.01 mg), while the nonspecific release at 37 °C was observed to be less than 5% of the DOXO-loaded amount (0.002 mg). This difference prompted us to test the therapeutic response on tumor cells, as shown in the next section.

### *In Vitro* Cytotoxicity (Trypan Blue Assay)

**Figure 5:**
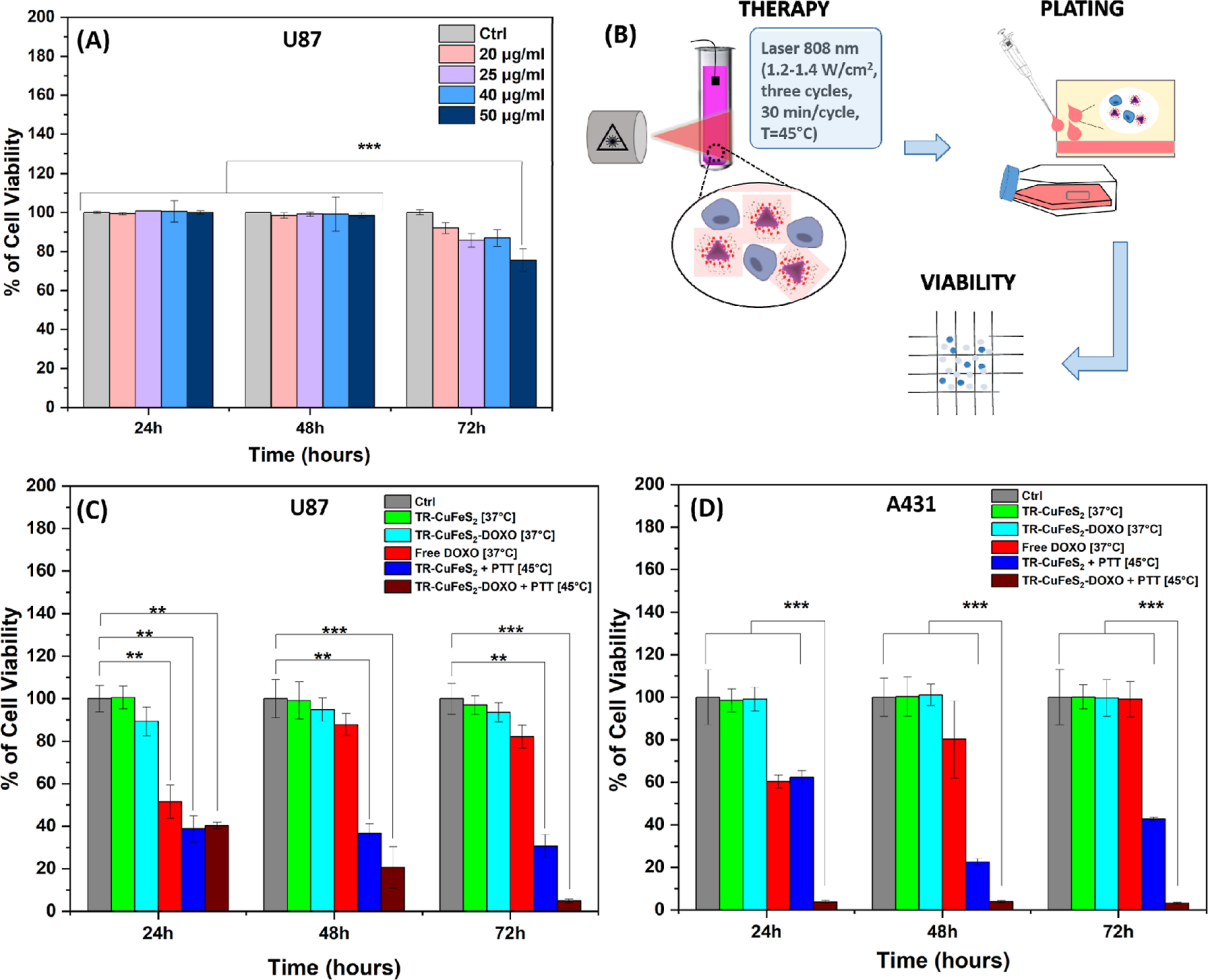
*In vitro* cell study. (A) Viability study of TR-CuFeS_2_ by trypan blue assay on U87 cell line at different NPs concentrations (20, 25, 40, and 50 μg/mL Cu). Values are presented as mean with error bars indicating the standard deviation (SD) for *n* = 3 independent experiments. The statistical analysis was performed using ANOVA with a Student–Newman–Keuls post hoc test. ***p = <0.001. (B) Experimental scheme for the evaluation of therapeutic effects: TR-CuFeS_2_ NPs (40 μg/mL Cu) were added to the cells, and PTT was applied. The viability by trypan blue was assessed on re-cultured cells at 24, 48, and 72 h. (C,D) Therapeutic viability results of PTT alone or in combination with DOXO release treatments on U87 and A431 cancer cells, respectively. Cell sample were grouped as follows: (i) control group: U87 cells not exposed to any treatment (Ctrl, in gray); (ii) material toxicity and nonspecific drug release groups: TR-CuFeS_2_ (no DOXO) kept at 37 °C (TR-CuFeS_2_ [37 °C], in green) and TR-CuFeS_2_-DOXO kept at 37 °C (TR-CuFeS_2_-DOXO [37 °C], in light blue); (iii) therapeutic groups: TR-CuFeS_2_ NPs exposed to PTT (TR-CuFeS_2_ + PTT [45 °C], in blue); TR-CuFeS_2_-DOXO NPs exposed to PTT (TR-CuFeS_2_-DOXO + PTT [45 °C], in dark red); and free DOXO at the same concentration released by the NPs (13% of the total amount) and kept at 37 °C (free DOXO [37 °C], in red). Values are presented as mean with error bars indicating the SD for *n* = 3 independent experiments. Statistical analysis was performed using a one-way ANOVA test (* 0.01 < *p* < 0.05; ** 0.001 < *p* < 0.01; ***p < 0.001) and Tukey’s HSD multiple comparison test.

Prior to validating the therapeutic effects of TR-CuFeS_2_ as a nanoplatform to combine PTT and controlled drug delivery, we investigated the range of biocompatibility of the TR-CuFeS_2_ NPs. In this study, 2D U87 malignant glioma cells (1*10^6^ cells) were incubated with TR-CuFeS_2_ NPs at different concentrations (20, 25, and 50 μg/mL Cu) for continuous NPs exposure at different time points (24, 48, or 72 h). The trypan blue assay showed a cell viability higher than 80% up to 48 h of incubation, at all studied concentrations, indicating no sign of toxicity (Figure 5A). At 72 h, a significant statistical difference with a reduction in cell viability was observed for 20, 25, and 50 μg/mL Cu experimental conditions. However, the viability for 20 and 25 μg/mL was higher than 80%, and therefore, we could define these doses as being nontoxic. At 50 μg/mL, the sign of toxicity (viability reduced to 70%) might be due to the slow release of Fe and Cu ions species from TR-CuFeS_2_ NPs in the intracellular acidic lysosomal environment, which may occur upon cell internalization. Indeed, the Fe-induced cell death through the ferroptosis process was shown to boost the toxic effects of PTT.^72,73^

### Combinatorial Phototherapy/CT Treatment Effects

Having assessed the biocompatibility range of the TR-CuFeS_2_ NPs, we next investigated the PTT and the chemotherapeutic effects of the NPs loaded with DOXO and the ones with no DOXO. For this experiment, we decided to use both brain tumor glioblastoma U87 cells and subcutaneous adenocarcinoma A431 cancer cells, the latter considered a golden standard in PTT *in vitro* studies. For this experiment, to one million cells in a pellet simulating a small tumor, the materials, either the TR-CuFeS_2_-DOXO (3.6 μg DOXO/mL/40 μgCu/mL) or the TR-CuFeS_2_ NPs (at 40 μgCu/mL), were added and exposed to PTT under an 808 nm laser (Figure 5B and see setup in Figure S10A and heating profiles in Figure S10B,C). By re-culturing the cells post-PTT exposure, the cell viability was monitored at different time points (24, 48, and 72 h) and compared to that of nontreated control U87/A431 cells that did not undergo any treatment (Ctrl) or to the viability of those cell samples that did receive the NPs material but were kept at 37 °C with no PTT exposure.

**Figure 6:**
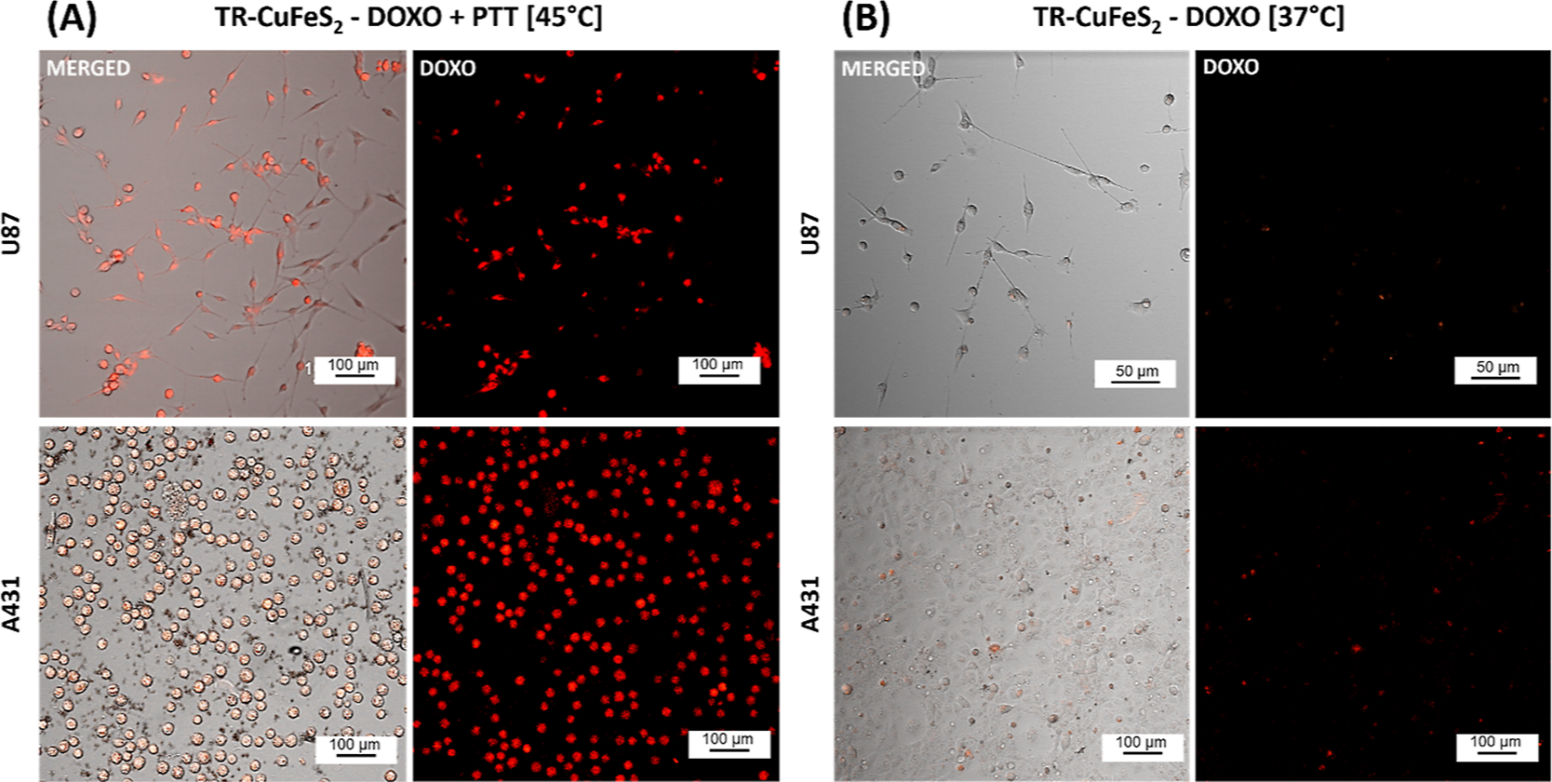
Intracellular uptake of DOXO. (A) Confocal study of U87 cells (upper raw) and A431 (lower raw) at 24 h post-treatment with TR-CuFeS_2_-DOXO and exposure to PTT at 45 °C. For both cell lines, we selected both the red confocal channel (right columns) with an excitation at 470 nm laser to image DOXO red signal and the merged channel given by the overlap of the bright contrast image with the red confocal channel (left columns) to better localize the red signal within the cells. (B) Images related to cells incubated with TR-CuFeS_2_-DOXO [37 °C] at 37 °C to evaluate the passive release of the drug from the NPs. (Scale bar: 50–100 μm).

**Figure 7:**
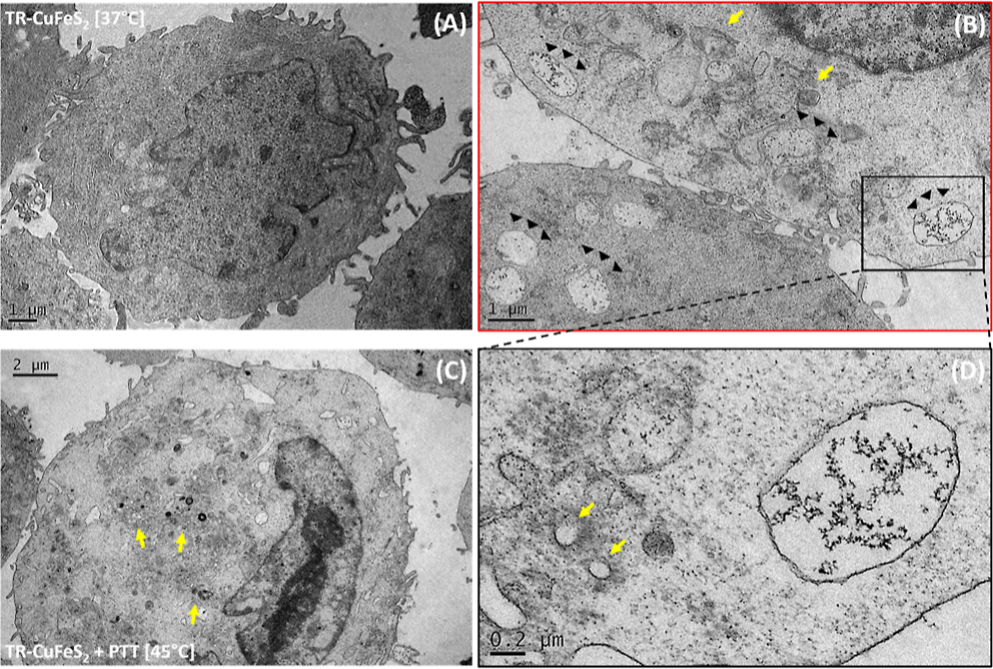
Representative TEM images of A431 cells upon NP exposure with or without laser irradiation. On the cell sample exposed to TR-CuFeS_2_-DOXO NPs + PTT [45 °C] (B–D) a significant number of intracellular vesicles similar to apoptotic bodies are present (yellow arrows). As shown in the enlargement images (red and black framed), NPs are visible as dark spots within endocytosis vesicles (black arrows). The typical appearance of a cell incubated with TR-CuFeS_2_ NPs and kept at 37 °C with no laser irradiation is shown in (A) with the cell body characterized by a physiological morphology, with no sign of sufferance.

The viability results showed that the combined effect of PTT and DOXO release by TR-CuFeS_2_-DOXO NPs caused the highest cytotoxicity against both tumoral cells (Figure 5C–D). In the case of U87, the cell viability gradually decreased to 41% after 24 h and reached 4% after 72 h. However, with A431 cells, the cell viability already significantly dropped 24 h post-treatment and remained below 5% for all time points of the experiments. This severe toxicity is ascribable to the synergic cell heating damage at 45 °C and the chemo-toxicity of the heat-mediated DOXO release. The DOXO release in both A431 and U87 cells was also confirmed by confocal images of the cells exposed to TR-CuFeS_2_-DOXO and PTT, which show a clear red fluorescent signal of the DOXO signal corresponding to the cells (Figure 6).On the contrary, in both cell lines, for the sample TR-CuFeS_2_-DOXO [37 °C] used to study the passive release of the drug, the viability was higher than 80% even after 72 h (Figure 5C,D, light blue bar) and the DOXO signal on the cells was also much less evident (Figures 6B and S11 for the free DOXO administered at the same dose), confirming that the nonspecific release from TR-CuFeS_2_-DOXO was only marginal at 37 °C. The application of PTT to the TR-CuFeS_2_ NPs with no DOXO loaded was able to just partially reduce the cell viability; the percentage of both A431 and U87 viable cells of the sample TR-CuFeS_2_ + PTT [45°] oscillated at about 40% up to 72 h, supporting the importance of synergic multiple treatments. To better localize TR-CuFeS_2_ NPs after PTT application and compare to cells treated with TR-CuFeS_2_ and kept at 37 °C, TEM images of cells were compared for these two samples. PTT effectively induced higher internalization of the TR-CuFeS_2_ NPs, as observed by the numerous endosomal vesicles filled with high-contrast NPs (Figure 7, black arrows). In the same images, apoptotic bodies (yellow arrows) are present, indicating cell suffering.

TR-CuFeS_2_ NPs [37 °C] exhibited 95–100% viability along the different time points, highlighting their nontoxic profile, and the TR-CuFeS_2_-DOXO at 37 °C were also nontoxic, suggesting that no significant drug leakage occurred within the first 72 h (Figure 5C,D).

The cytotoxicity data were also compared to those obtained when incubating the cells with free DOXO at the same drug dose released (0.48 μg) by TR-CuFeS_2_-DOXO NPs and under exposure to either a warm water bath set at 37 °C [DOXO (37 °C) in Figure 6B] or to a bath set at 45 °C [DOXO (45 °C)] that is the PTT temperature for the same time PTT treatment (Figure S12).

Not having seen any difference within these two groups [TR-CuFeS_2_-DOXO + PTT (45 °C) and DOXO (45 °C)], it may appear that the effect of ROS production for the TR-CuFeS_2_-DOXO + PTT [45 °C] sample in the viability is marginal. With U87 as well as A431, the sample with DOXO [37 °C] showed that the single effect of the drug was able to induce an acute cytotoxicity in the cells after 24 h (cell viability of 50%), which was then recovered to about 80% after 72 h. However, the data for free DOXO [45 °C] (Figure S12) showed a comparable cell toxicity to that of TR-CuFeS_2_-DOXO + PTT [45 °C] (Figure 5C,D), indicating that the heat and the DOXO released are the major causes of synergic cell damage. We also evaluated the ROS production intracellularly. The exposure to TR-CuFeS_2_ NPs and 5 min laser at 1.5 W/cm^2^ (these conditions were chosen for compatibility with the ROS test on cells) was inducing an increase in fluorescent intensity of DCF related to ROS production. This mean intensity was statistically significant with respect to the basal intensity recorded on only A431 cells. Instead, the mean intensity of a cell sample incubated with only NPs was not significant with respect to A431 cell only (Figure S13). Therefore, these data suggest that the heat damage together with intracellular ROS production under laser is responsible for the cell toxicity. In addition to that, the drug release also contributes to more effective therapy when considering TR-CuFeS_2_-DOXO + PTT [45 °C].

## Conclusions

In summary, our work reports the development of a thermoresponsive polymer-functionalized chalcopyrite NP system (TR-CuFeS_2_ NPs) for use in combined PT–CT applications. This novel hybrid nanomaterial was produced by means of a simple ligand exchange reaction wherein a customized multi-amine-bearing TR-polymer P(DEGMA-*co*-OEGMA), synthesized *via* a photo-atom transfer radical polymerization technique, was grafted to the surface of CuFeS_2_ NPs. We corroborated the colloidal stability, heating performance, as well as the ROS generation feature as a function of the NPs’ dose and laser power densities. As a nanocarrier, TR-CuFeS_2_ NPs can efficiently load the anticancer drug, DOXO (up to 90 μg DOXO per mg Cu) and release it in a heat-dependent manner when irradiated at mild NIR-light conditions. Furthermore, our cytotoxicity study indicated that these NPs, at a dose 40 μg/mL [Cu] and under laser (808 nm, 1.2 W/cm^2^) irradiation, can heat up the cell media from 37 °C to a therapeutic temperature of 45 °C. This heat produces synergistic damage effects with the drug release (heat-mediated) on squamous (A431) cancer cells as well as on Glioblastoma (U87) cells. Despite the well-known limited penetration depth of NIR light, the choice to use brain cancer cells is justified by the possibility to soon exploit implantable waveguide light-emitting diodes coupled to a tapered optical fiber. As shown in optogenetics for deep brain optical stimulation,^74,75^ the tapered fibers are able to deliver light in scattering tissues, like the brain, at a penetration depth up to 2 mm, thus making PTT doable in brain tumors also. Furthermore, having used such a low copper dose NPs, no toxicity of the TR-CuFeS_2_ NPs material was recorded for up to 48 h. Based on these overall results, we can state that our novel TR-CuFeS_2_ nanoplatform represents a promising future candidate in a clinical setting, as an adjuvant therapy to surgery, in order to eliminate the surrounding tumoral areas remaining after glioblastoma removal. In perspective, it is also worth mentioning that the CuFeS_2_ NPs offer the possibility to insert a 64-Cu radioisotope into the crystal structure by cation exchange reactions,^47^ and these cation exchange protocols on TR-CuFeS_2_ NPs will enable combining phototherapy, CT, and also internal RT together within one single platform.

## Supporting Information Available

Materials and methods, DLS measurement for LCST of TR-polymer, additional TEM image of TR-CuFeS_2_ NPs, XPS spectra, additional extinction spectra of the TR-CuFeS_2_ NPs, set up image for PT conversion efficiency determination, photoluminescence spectra for ROS determination, zeta potential of TR-CuFeS_2_ NPs before DOXO loading and hydrodynamic size spectra weighted by intensity, volume, and number for TR-CuFeS_2_ DOXO NPs, DLS traces for stability study of TR-CuFeS_2_ and TR-CuFeS_2_-DOXO NPs in physiological conditions, photo of laser 808 nm setup used in our study, heating profile curves of U87 cells with pristine TR-CuFeS_2_ NPs, additional confocal images, cell viability test of controlled tumor cells, and proof of ROS production by fluorescent intensity study (PDF)
