# Supplementary material for: CuFeS2 Nanoparticles Functionalized with a Thermoresponsive Polymer for Photothermia and Externally Controlled Drug Delivery
Source: ACS Appl Mater Interfaces. 2023 May 3;15(19):22999–3011. doi: 10.1021/acsami.3c03902 (PMC10197081; doi:10.1021/acsami.3c03902)
Supplement: Supplementary file 1 — am3c03902_si_001.pdf [file am3c03902_si_001.pdf]

Supporting Information

$CuFeS_2$ Nanoparticles Functionalized with a Thermo-Responsive Polymer for Photothermia and Externally Controlled Drug Delivery

John S. Conteh,[1,2] Giulia E. P. Nucci, [1,2] Tamara Fernandez Cabada, [1] Binh T. Mai, [1] Nisarg Soni, [1] Francesco De Donato, [1] Lea Pasquale, [1] Federico Catalano,[1] Mirko Prato,[1] Liberato Manna[1] and Teresa Pellegrino[1]*

1 Italian Institute of Technology, via Morego 30, 16163, Genoa, Italy

2 Dipartimento di Chimica e Chimica Industriale, Università di Genova, Via Dodecaneso 31, 16146, Genoa, Italy

*Corresponding author: Teresa.Pellegrino©iit.it

Experimental Section

Materials

N-Hydroxysuccinimide (NHS, Aldrich, 98%), Triethylamine (TEA, Merck, 99%), 2 – Bromoisobutyrylbromide (2-BiBA, Aldrich, 98%), Tetraethylenepentamine (TEPA, Aldrich), were all used as purchased. Oligoethylene glycol methyl ether methacrylate (OEGMEMA, M_w 500 g mol^{-1}, Aldrich, 99%) and Diethylene glycol methyl ether methacrylate (DEGMEMA, Aldrich, 95%) monomers were purified by passing through an Alumina loaded 24 mL Norm-Ject plastic syringe fitted with 0.45 µm Sartorius filter to remove inhibitors. N-hydroxysuccinimidyl bromoisobutanoate ester (NHS-Ester), Copper bromide ($CuBr_2$) and Tris[2-(dimethylamino)ethyl]amine (Me_6TREN, 97%) used as catalyst were purchased from Sigma-Aldrich and used as received . All solvents were of highest purity (≥ 99%) and used as purchased from Sigma-Aldrich. Doxorubicin hydrochloride (DOXO, Aldrich, Europian Pharmacopia Reference Standard).

Cell culture

All of the cell culture reagents were purchased from Gibco. U87 MG glioblastoma cells were provided by Dr. Emilio Ciusani from Carlo Besta Neurological Institute from Milano (Italy). A-431 were purchased from ATCC. Cells were cultured on 2D model in Dulbecco's Modified Eagle's Medium (DMEM, high glucose) supplemented with 10% fetal bovine serum (heat inactivated FBS), 2% penicillin−streptamycin (10000 U/mL), and 1% L-glutamine (200 mM) at 37 °C, 5% CO2, and 95% relative humidity. NP experiments were performed at cell passage number between 24 and 26.

General Characterization Techniques

The hydrodynamic sizes (D_h) of the NPs were determined using a Zetasizernano dynamic light scattering (**DLS**) instrument. For each measurement, 500 µL of the sample in appropriate solvent was piptted into a disposable cuvette before triplicate readings were made following an equilibration time of 1 min per reading. To obtained all the Transmission Electron Microscopy (**TEM**) images a JEOL JEM 1400Plus electron microscope was used. This instrument is equipped with a LaB_6 crystal thermionic electron source having an acceleration voltage of 120 kV, and an Orius CCD Camera purchased from Gatan company, USA. To do the measurement, samples either from water or from chloroform solutions were dropped onto a carbon coated copper grid and allowed to dry before being viewed under the miscroscope. The polyemr and initiator were characterized by Proton Nuclear Magnetic Resonance (**^{1}H NMR**) spectroscopy using a Bruker 400 MHz BBI spectrometer with deuterated DMSO as solvent at 25°C. To evaluate the success of the surface polymer ligand exchange Fourier Transformed Infrared (**FTIR**) spectroscopy was performed, using a Vertex 70V Bruker instrument having an attenuated total reflectance (ATR) configuration coupling a MIRacle ATR (PIKE Technologies). Visible-Near-InfraRed (**NIR**) absorption spectra were recorded using a Cary5000 spectrometer at a wavelength range of 400 – 1200 nm. For the Doxorubicin quantification, a **UV – Visible** Cary300 spectrometer was used with the spectra recorded at a wavelength range of 400 – 800 nm. For elemental quantifications, a ThermoFisher CAP 6000 series Inductively Coupled Plasma Optical Emission Spectrometer (**ICP-OES**) was used. For these measurements, 10 µL of the sample was added to 1.0 mL of aqua regia for an overnight digestion. The sample solution was then diluted to 10 mL with Milli-Q water and then filtered through a 0.45 µm PTFE membrane before the analysis. For the phothermal and ROS mesasurements, a Roithner **laser source** (808 nm, spot size diameter of 0.56 cm, focal length 18 cm at power density in the range between 0.33 and 1.5 W.cm^{-2}) was used.

Synthesis of the Amine terminated ATRP initiator ligand (TEPA-BiBA)

To synthesize our customized ATRP inintiator, we pre-synthesized an NHS ester derivative that later underwent aminolysis with Tetraethylenepentamine, as described below.

NHS Ester derivated of 2 – Bromoisobutyryl Bromide (NHS-BiBA ester) synthesis

To a 50 mL round bottom flask containing (2.3g, 0.01mol) N-Hydroxysuccinimide (NHS) ester dissolved in 30 mL of Chloroform, 3.3 mL (0.012 mol) of Triethylamine (TEA) was added and maintained at 0°C with ice while magnetically stirring. Over a 20 minutes period, drops of 2 – Bromoisobutyryl Bromide (1.4 mL, 0.01 mol) were added to the mixture and stirred further for 20 minutes. The solution was washed with two separate 16 mL aliquots of ice-cold water and saturated brine, dried with $MgSO_4$ and filtered. Then, the chloroform solution containing the crude NHS-ester product was concentrated to a volume of 6.0 mL under vacuum. Next, 4 mL of Ethyl acetate and 30 mL of hexane solutions were added under stirring and kept at 0°C, for 3 hours. The crude white NHS-ester precipitate obtained was filtered, recrystallized and dried in a vacuum oven overnight before NMR characterization.

ATRP initiator (TEPA-BiBA) synthesis by NHS ester aminolysis

The aminolysis reaction of our synthesized NHS-BiBA ester with Tetraethylenepentamine (TEPA) that resulted to our customised ATRP Bromide derivate initiator was carried out at room temperatures. Briefly, to a 40 mL vial containing 18 mL Chloroform, 189.7 µL (0.001 mol) of TEPA was added. To this solution, 2 ml of NHS ester (0.3382 g, 0.0011 mol) in Chloroform was added at a rate of 0.5 mL per 10 min while still shaking (at about 600 rpm) and left to react overnight at room temperature. The reaction mixture was then filtered through a whatman filter paper to remove the precipitated NHS by-product and the recovered solution was later completely evaporated to dryness to obtain the gold-yellow gel-like product (TEPA-BiBA) that was subsiquently confirmed by TLC and ^{1}HNMR characterizations.

TR-polymer Synthesis

The synthesis of the thermoresponsive copolymer to be grafted on the surface of $CuFeS_2$ NPs was done using a Photo atom transfer radical polymerization technique (photo-ATRP) technique. With this techque, less amount of copper catalyst is required compared to the classical ATRP, which means less purification cost and time. For a successful photo-ATRP polymerization, the ratios of [Monomer]: [Initiator]: [Me_6TREN]: [$CuBr_2$]; were tuned to 20:1: 0.08: 0.04 respectively. To achieve a TR-polymer with an LCST of 42°C we tuned the amphiphilic Diethylene glycol methyl

ether methacrylate [DEGMEMA] to hydrophylic oligoethylene glycol methyl ether methacrylate [OEGMEMA] monomer ratios to 88:12. In details, to a 40 mL vial was pipetted 2420 µL of 0.1 mg/mL ATRP initiator in DMSO followed by the addition of another 4.0 mL DMSO solution containing inhibitor free DEGMEMA (2325 µL, 0.126 mmol), OEGMEMA (796 µL, 0.0172 mmol) and 16 µL Me_6TREN ligand. After which, the vial was sonicated for 30 s and purged with Nitrogen for 15 min through a needle piersed into the vial septum cap . In another separate 3 mL vial, we prepared a 2 mL DMSO solution containing 6.4 mg $CuBr_2$. This was also similarly purged with Nitrogen before slowly (within a minute) injected into the initial 40 mL vial while still under Nitrogen. The resulting mixture was then photo-polymerized in a cold room (5°C) for 6 h using a UV lamp (ʎmax 365 nm) sited on an orbital shaker at a speed 100 rpm for 6h, after which the reaction was quenched by air exposure and the viscous blue coloured crude product was diluted with excess THF solvent (roughly 50 mL). To enable the removal of the copper catalyst, the diluted polymer solution was filtered through an Aluminum Oxide packed column and afterwards concentrated by rota-evaporation to a final volume of ca. 10 mL. This concentrated polymer solution was precipitated with cold diethyl ether (3 folds excess by volume) and centrifuged at 3000 rpm for 10 min (3 times) and later vacuum oven dried for 24 h. The 2.3 g of TR-polymer (*ca.* 70% yield) obtained herein was dissolved in Chloroform for use in the subsequent ligand exchange step.

Synthesis of $CuFeS_2$ NPs

Chemicals: CuI 99,5%, 1-Octadecene 90%, Fe(acac)3 99%, 1-Dodecanethiol (DDT) 98%, Oleylamine (OLAM) 70%, Chloroform anhydrous 99%, 2-Propanol anhydrous 99,5 % were purchased from Sigma Aldrich. The Tri-n-octylphosphine (TOP) 97% was purchased from Strem Chemicals.

In a glovebox under nitrogen, a solution of 1mmol of CuI (190 mg) and 1 mmol of TOP (0.46 mL) in 1 mL of ODE was prepared. The mixture was heated under stirring at 80°C, to yield a transparent solution. Meanwhile, in a 50 mL tri-neck flask, 1 mmol of $Fe(acac)_3$ (353 mg) was dissolved in 6 mL of ODE. The flask was connected to the Schlenk line and sealed to be exposed to vacuum at 80°C for 1 hour. Next, after switching the line from vacuum to N_2 atmosphere, the solution of CuI:TOP was quickly injected into the flask and the temperature was set at 280°C (heating rate of 15 °C/min). The Sulphur precursor solution was prepared in a separate 25 mL tri-neck flask by addition of DDT (2 mL) in 4 mL OLAM, followed by a vacuum cycle at 80°C for 30 minutes, then switching to nitrogen atmosphere and heating the solution at 160 °C (heating rate of 15 °C/min) to yield a clear and yellow solution. Next, with a glass syringe (14 gauge

needle), the hot DDT solution (3 mL) was injected into the Cu and Fe precursors solution in the flask kept at 280°C. Just after the injection, a drop in temperature up to 230°C was recorded and the solution turned from red to a dark purple color. The reaction temperature was promptly set at 270°C and kept at this value for 15 minutes to enable the growth of the nanocrystals. The reaction was quenched by removing the heating mantle and directing a flow of compressed air onto the flask. 5 mL of Chloroform were then added to the reaction mixture to facilitate the transfer of the viscous solution with a plastic syringe, into a 40 mL vial (formerly closed into the glovebox to be maintained under nitrogen). The final solution was washed inside the glovebox with anhydrous solvents: 5 mL of 2-propanol was added to the reaction solution and centrifuged at 3500 rpm for 5 minutes. The nanocrystals were resuspended in 5 mL Chloroform and washed again with 2-propanol as described. The NCs were finally suspended in chloroform and centrifuged at 1000 rpm for 5 minutes, and the solution was transferred in a new vial by means of a micro-pipette carefully avoiding to collect the precipitated fraction. The final sample was characterized with TEM and ICP-OES analyses.

Ligand Exchange (ratio of 50 polymer ligand/nm^2)

The synthesized TR-polymer was then "grafted to" the surface of our pristine $CuFeS_2$ NPs by means of a simple ligand exchange reaction. In a typical exchange procedure, 78 µL of $CuFeS_2$ nanoparticles in chloroform (0.5 mg, 6.4 mgCu/mL , 13 nm) was added to a 20 mL vial containing 2.5 mL of Chloroform. This was followed by a subsequent addition of 600 µL of the amine terminated TR-polymer (60 mg, 0.1 g/mL). Then, the vial was sonicated for 30 s, foiled and left gently shaking over two nights. After the exchange period, the resulting crude product (TR-$CuFeS_2$ NPs) was cleaned by precipitation with 33 mL cold diethyl ether and later centrifuged at 4300 rpm for 12 min. To ensure complete removal of the chloroform solvent which is immiscible with water, the obtained precipitate (TR-$CuFeS_2$ NPs) were re-dispersed in 2.5 mL THF and precipitated as before. Following an hour (1 h) drying under nitrogen flow to remove all ether solvent, the TR-polymer coated NPs (TR-$CuFeS_2$) were then dispersed in 5 mL water and washed by 3 cycles of centrifugal filtration (MWCO 100 kDa, 2200 rpm, 30 min). This was done to further clean and concentrate the sample to about 200 µL volume before characterization.

Comparative X-ray Photoelectron Spectroscopy (XPS) Characterization of TR-$CuFeS_2$ NPs

X-ray photoelectron spectroscopy (XPS analysis) was carried out with a Kratos Axis Ultra DLD spectrometer using a monochromatic Al Kα source, operated at 20 mA and 15 kV. Specimens for

the XPS measurements were prepared by drop casting few microliters of a concentrated NC solution onto silicon substrates.
Survey scan analyses were carried out with an analysis area of 300 × 700 μm and a pass energy of 160 eV. High resolution analyses were carried out over the same analysis area at pass energy of 20 eV. The Kratos charge neutralizer system was used on all specimens. The binding energy scale was calibrated by setting the carbon 1s component due to C-C bonds to 284.8 eV. Spectra were analyzed using CasaXPS software (version 2.3.25).

Protocol for TR-$CuFeS_2$ NP molar extinction coefficient determination

Here the molar extinction coefficient was determined by measuring the absorption spectra of six different TR-$CuFeS_2$ NP solutions in Milli-Q water and applying the Beer- Lambert law:

$A\ (\lambda) = \varepsilon \times l \times c$ ……… (1)

Where, A refers to the absorbance at a specified wavelength λ, ε is the molar extinction coefficient in $M^{-1}cm^{-1}$, l is the optical path length of the cuvette in cm and c is the molar concentration of NPs. The NPs concentrations were determined from elemental ICP-OES analysis, by measuring the Fe and Cu amount taking into consideration the volume of a single NP. The Absorption spectra of TR-$CuFeS_2$ NPs at concentrations ranging from 1.08×10^{-8} to 1.08×10^{-7} in water were recorded (Figure S4A) and the absorbance values at wavelengths of 499, 808 and 1000 nm were plotted with respect to the NP concentrations (Figure S4B). The molar extinction coefficients (Ɛ) summarized in Table S1 were calculated from the slopes of the linear fit of these plots and knowing the cuvette's path length using the equation Ɛ =Slope/l where "l" is the path length of the cuvette (0.3 cm).

<u>Photothermal Conversion efficiency Experimental Protocol</u>

To determine the photothermal conversion efficiency of TR-CuFeS2 NPs a protocol and the experimental setup (Figure S5A) previously reported by some of us was adopted. [1] Here, an aqueous solution of the NPs or plain water injected in a glass cuvette contained in a self-made irradiation chamber (see design description in Figure S5A), were irradiated with laser in vacuum while measuring their heating profiles overtime. After reaching a temperature plateau, the laser was switch off and the cooling curve of the NP was used to calculate the photothermal conversion. In details, prior to irradiation, a 70×10^{-2} mmbar vacuum was applied to the irradiation chamber by connecting it to a Schlenk line. Then 1 mL aqueous solution of TR-$CuFeS_2$ NPs (0.12 mg/mL Cu; Absorbance at 808 nm = 0.574, mass of TR-$CuFeS_2$ (g) = 0.9661) was placed into the quartz

cuvette (path length = 1 cm). A thermal-probe was dipped through the glass tube into the cuvette to touch just the upper part of the NP solution. Using a laser 808 nm device (laser power 0.753 W, spot size 0.2424 cm^2), the NPs solution was irradiated for about 25 minutes before being switched off when the solution temperature reached a plateau (ca. 54.6 °C) starting from a temperature of about 22.7°C. The thermal profile of the cooling processed was also recorded using the thermoprobe to yield the representative heating and cooling curve shown in (Figure S5B, red plot). By employing the same set up and laser conditions, the heating-cooling cycle of 1 mL deionized water was also obtained (Figure S5B, black plot).

Using the NP's cooling curve and employing the total energy balance for the system as expressed by the equation (1), the photothermal conversion efficiency of TR-$CuFeS_2$ NPs was calculated following a protocol we reported in literatures and well described in the ESI of the work reported by Gosh et al. [1,2] Details of the equations and data used in the photothermal conversion efficiency calculation are provided here.

The system's total energy balance is expressed by the equation

$$\sum_i m_i Cp_i \frac{dT}{dt} = Q_{NC} + Q_{Dis} - Q_{Cond} \qquad (1)$$

where *m (g) and Cp* ($J \cdot g^{-1} \cdot K^{-1}$) are the mass and specific heat capacity, *T* (K) is the temperature of the solution, Q_{NC} (W) is the energy input by NCs under laser irradiation, Q_{Dis} (W) is the energy input of the water contained in the quartz cuvette and Q_{Cond} (W) is the heat conduction from the cuvette to the surrounding.

Q_{NC} is calculated using the equation

$$Q_{NC} = I\left(1 - 10^{-A808}\right)\eta \qquad (2)$$

where *I* (W) represents the laser power used during irradiation, A808 is the absorbance of NPs solution at the irradiation wavelength (808 nm) and η is the conversion efficiency from incident laser energy to thermal energy.

Q_{Cond} can be determined by

$$Q_{Cond} = hS\left(T - T_{amb}\right) \qquad (3)$$

where *h* is the heat transfer coefficient ($W \cdot cm^{-2} \cdot K^{-1}$), *S* is the surface area (cm^2) and T_{amb} the ambient temperature (K). Q_{Cond} is increasing along with the increase of the temperature during irradiation step and will rise to a maximum when the heat input is equal to the heat output.

$$Q_{NC} + Q_{Dis} = Q_{Cond-max} = hS(T_{Max} - T_{amb}) \qquad (4)$$

The heat efficiency can be obtained by substituting equation (2) in equation (4) obtaining

$$\eta = \frac{hS\,(T_{max} - T_{amb}) - Q_{Dis}}{I(1 - 10^{-A808})} \quad (5)$$

To obtain hS, a dimensionless driving force temperature (Θ) is introduced using the maximum system temperature (T_{max})

$$\theta = \frac{T - T_{amb}}{T_{Max} - T_{amb}} \quad (6)$$

and a sample system time constant

$$\tau_s = \frac{\sum_i m_i C_{p,i}}{hS} \quad (7)$$

Substituting equation (6) and (7) in equation (1) and rearranging, taking into account that during the cooling stage $Q_{NC} + Q_{dis} = 0$, the equation is reduced to

$$dt = -\tau_s \frac{d\theta}{\theta} \quad (8)$$

By integrating equation (8) we obtain

$$t = -\tau_s \, ln\,(\theta) \quad (9)$$

From equation (9), the system time constant (τ_s) is obtained from the slope of the linear fit of time/-ln(θ) during the cooling step of TR-$CuFeS_2$ NPs solution (see Figure S5C)

By rearranging equation (7) and inserting this $\tau_s = 442.69$, *hS* was calculated to be 0.02 W K^{-1}.

Q_{dis} was determined from the cooling stage after same laser treatment of the quartz cuvette filled with 1 mL of water. Q_{dis} was finally calculated following equation (10)

$$Q_{dis} = hS(T_{MaxWater} - T_{amb}) \quad (10)$$

resulting in 0.037 W, since $T_{max\ water}$-T_{amb} was 1.87 K.

Finally, the photothermal efficiency (*η)* of TR-$CuFeS_2$ NPs was determined from equation (5) using data shown in Table S2:

$$\eta = \frac{hS\,(T_{max} - T_{amb}) - Q_{Dis}}{I(1 - 10^{-A808})} \quad (5)$$

Photothermal study of TR-$CuFeS_2$ NPs

In a typical experimental protocol, an Eppendorf containing 500 µL of TR-$CuFeS_2$ NPs (0.05 mgCu/mL in Saline pH 7.4) was irradiated for 15 minutes using a laser 808 nm source operated at varying power densities (0.33 – 1.5 W/cm^2, spot size 0.56 cm diameter). With a probe inserted in the TR-$CuFeS_2$ solution, the temperature was measured and the heating profile obtained.

<u>ROS Quantification of TR-$CuFeS_2$ under Laser 808 nm Irradiation</u>

To quantify the ROS production as a function of laser power density, four (4) Eppendorf vials containing each 0.5 mL TR-$CuFeS_2$ in saline solution (0.05 mgCu/mL) were irradiated with a 808 nm laser source at power densities of 0.33, 0.5, 1.0 and 1.5 W/cm^2 for 15 minutes. Just after irradiation, 200 µL DCFH-DA dye (5 µM DCFH and 0.5 units/mL Horseradish peroxidase) was added to each NP solution and the Eppendorfs were coated with aluminium foil and incubated for 2.5h in dark conditions. The solution was then filtered on centrifuge filters (Amico tube, MWCO 100 kDa, 5000 rpm, 5 min) to separate the NPs (upper filter) from the filtrate solution (bottom of the filter) and on the latter the fluorescence was measured (Excitation 485 nm, emission 500 nm) (Figure S6A). In a similar manner, the dependence of ROS generation on NP dose was studied by irradiating 0.5 mL TR-$CuFeS_2$ in saline solutions at different nanoparticles concentration in the range 0.02 - 0.1 mgCu/mL for 15 minutes with a 808 nm laser source operated at 1 W/cm^2. The PL spectra (Figure S6B) obtained after dye addition, incubation and filtration were used to quantify the amount of ROS generated by the TR-$CuFeS_2$ NPs at the different nanoparticle doses.

The photoluminescence (PL) intensities values at 523 nm were converted in ROS amount expressed in nM [H_2O_2] on a calibration curve following a method previously reported.[3,4] For the calibration plot, 1.218 mg of 2,7-Dichlorofluorescin diacetate (DCFHDA) were dissolved in 2.5 mL ethanol. To deacetylate the DCFHDA to DCFH, 10 mL of 0.01M NaOH solution were added to the DCFHDA solution and the mixture stored in the dark at room temperature for 30 minutes. Next, 6.25 mL of this hydrolyzed DCFH solution was neutralized with 243.8 mL of 25 mM phosphate buffer (pH = 7.2) that contains 2.5 mg enzyme Horseradish peroxidase (HRP, Type I, 50 unit/mg) to yield 250 mL solution at 5 µM of DCFH and 0.5 units/mL of HRP.

For the calibration plot (Figure S6C), starting with initial 30% (w/w) peroxide (H_2O_2) purchased from Sigma Aldrich, a series of solutions at concentrations of 1.36×10^{-7}, 1.82×10^{-7}, 2.27×10^{-7}, 2.54×10^{-7} , 2.82×10^{-7} and 3.82×10^{-7} M 1.5×10^{-6} to 4.2×10^{-6} M respectively were prepared. These solutions contained each also 3 mL of the 5 µM DCFH dye/HRP solution previously prepared. After the dye addition, these mixtures were kept in the dark for 2.5 h to equilibrate before measuring their fluorescence using a Cary eclipse spectrophotometer, at an excitation wavelength of 485 nm and emission of 523 nm. The PL intensity was plotted as a function of the H_2O_2 concentration and the curve was used to estimate the amount of ROS species by the PL intensity of the TR-$CuFeS_2$ NPs solutions under various laser irradiation conditions.

DOXO Loading on TR – $CuFeS_2$ NPs

The DOXO encapsulation to the TR – $CuFeS_2$ NPs was performed in Milli-Q water. Specifically, to a TR – $CuFeS_2$ NPs (0.1 mgCu) dissolved in 5 mL Milli-Q water (pH 7.4), an aliquot of 50 µL

free DOXO (0.05 mg, 1 mg/mL) in water was added and the solution was left shaking on the orbital shaker over the 6 hr. This solution was washed 3 times on a 100 kDa filter tube (5 mL) at 1800 rpm for 20 min using Saline solution (pH 7.4) to remove unloaded DOXO. The DOXO loaded NP (TR – $CuFeS_2$-DOXO NPs) was concentrated to about 200 µL volume for later study.

TR-$CuFeS_2$/DOXO NPs stability study

To investigate the NP's stability in saline media (0.9%), saline solutions of the TR-$CuFeS_2$ and TR-$CuFeS_2$-DOXO NPs (0.04 $mg_{[Cu]}$/mL) were prepared and kept at room temperature for a period of 8 days to measure, every day, the hydrodynamic sizes intensities using a DLS instrument. Visual inspection of the NP solutions and sample photos were captured overtime.

Quantification of loaded DOXO amount on TR – $CuFeS_2$-DOXO NPs

The quantification of the loaded DOXO amount was achieved using EDTA release protocol. In brief, to a 10 µL TR – $CuFeS_2$-DOXO NPs(0.1 mgCu/mL) solution in saline, 40 µL (0.5M) EDTA solution and 20 µL HCl(0.001M) were added and the mixture incubated in a water bath set at 60°C for 1 hour to induce the phase precipitation of TR – $CuFeS_2$ NPs. To separate this shrinked TR-$CuFeS_2$ NPs from the release DOXO after the heat treatment, the solution was centrifuged at 4000 rpm for 5 minutes to bring down the NPs and the collected supernatant was UV characterized at 485 nm wavelength. By matching the obtained absorbance value to a prepared standard DOXO calibration plot (ranging from 0.01 to 0.06 mgDOXO/mL), the total amount of loaded DOXO was calculated.

Quantification of release DOXO amount from TR – $CuFeS_2$-DOXO NPs

To quantify the amount of non-specific and controlled release of DOXO from our TR – $CuFeS_2$ -DOXO NPs, two sets of NP solutions were exposed to either water bath or laser 808 treatments respectively. In the non-specific study done to mimic the release of the drug at physiological condition, we expose the TR – $CuFeS_2$-DOXO NPs solution to a water bath maintained at 37°C (less than the LCST of TR – $CuFeS_2$-DOXO NPs). Here, 400 µL saline solution of TR – $CuFeS_2$-DOXO NPs (0.1mgCu/mL/ 9.1µgDOXO/mL, pH 7.4) was added to a 500 µL Amicon filter and transferred to the water bath where it was kept for 1 h. Next, the sample was cooled to room temperature and centrifuged twice (5000 rpm, 13 min) to wash off the released DOXO, using fresh saline solution. At the end of the second wash, the in-filter portion was made up to 400 µL mark by saline solution addition before collecting 10 µL aliquot from it for DOXO amount quantification *via* the EDTA release protocol described earlier. This process was repeated 6 times (6 cycles of 1h heat treatment each) on the same starting sample. In a similar manner, the control release

study was carried out but with laser (808 nm, 1.0 W/cm^2) instead of the waterbath. In this case, the TR – $CuFeS_2$-DOXO NPs solution was irradiated to attain a temperature of 43°C, which is above the LCST of the surface grafted TR-polymer. By noting the deference in sample DOXO amount before and after treatment, the cumulative percentage DOXO release profiles per time for the two studies were obtained.

***In Vitro* Cytotoxicity Assay**

U87 MG and A-431 cells were plated in 24-multiwell plates at a cell density of 15000 cells/well (72 h cytotoxicity assay), 30000 cells/well (48 h cytotoxicity assay) and 60000 cells/well (24 h cytotoxicity assay) to allow them reach a confluence state at the end of the test. NPs were added 24 h after cell seeding in order to ensure the good health status of the cells. A concentration range of 20-50 µgCu/ml of TR-$CuFeS_2$ NPs in the a complete cell culture medium was tested on cells. Control cells did not receive treatment with NPs. NPs were incubated for 24 h, 48 h or 72 h time period. After the incubation periods, a cytotoxicity assay was conducted using the Trypan Blue exclusion test. The experiment was done in triplicate (n = 3), in three independent experiments, and the mean value plotted with an error bar to represent the standard deviation. Bright field pictures were taken also in order to confirm by morphological inspection the results from cytotoxicity trypan blue test. Pictures were taken at different time points using a Nikon A1+ confocal microscope system.

Treatment effect in i*n Vitro* Assay

U87 MG and A-431 cells were cultured in Dulbecco's Modified Eagle's Medium (DMEM, Sigma-Aldrich) supplemented with 10% heat-inactivated FBS. 2mM L-glutamine, 1% penicillin/streptomycin in T-75 flask. Once confluence was reached, cells were detached and 1*10^6 cells was counted using a Nucleocounter to be used for each experimental condition. Then, cells in pellet were re-suspended in 60µl of complete medium alone, or with free DOXO drug (0.48 µg) or with TR-$CuFeS_2$ NPs (40 µg Cu/ml) or with or with TR-$CuFeS_2$ NPs-DOXO (40 µg Cu/mL/ 3.6 µg DOXO/mL). The mixture was then transferred into a UV-sterilized glass probe. The samples that had to undergo the laser treatment were exposed to three cycles of PTT (30 min each, T=45°C, power density=1.2-1.4 W/cm^2). All the others were kept in a cell culture incubator at 37°C for one hour and a half. Then, for all the samples, cells were collected and plated back into T-25 flasks without washing out the NPs. The cell viability was assessed at 24 h, 48 h and 72 h after the treatment by means of Trypan Blue exclusion method. The experiment was performed in triplicate (n = 3) and the mean value plotted with an error bar to represent the standard

deviation. Confocal imags were taken using a Nikon A1+ confocal microscope system after 24 h from the treatment to show the internalization of Doxo within the cells.

Intracellular Reactive Oxygen Species Production: A431 cancer cells were seeded into a 24-well plate (2*105) for 24 h to allow the cells to adhere to the wells. TR-$CuFeS_2$ NPs (40 µg.mL-1 Cu) were added directly in the cells media and the cells were incubated for 6h at 37°C. The control cells were incubated at 37°C for the same time with only TR-$CuFeS_2$ NPs and no laser exposure. One group was used for photo-irradiation with the cell pellet exposed for 5 minutes to the laser at a power density of 1.5 W/cm^2. Subsequently, cells were re-cultured in 2D and, after incubation for 6 hours, they were washed with phosphate buffered saline (PBS, pH 7.4). Next, new media containing the dye DCFHDA was added to the cells at the concentration of 10 µM and samples were incubated for 30 minutes at 37°C. Fluorescent intensity was determined with excitation at 485 nm and emission at 520 nm using a plate reader.

Statistical analysis

The experiments were done in triplicate (n= 3) and statistical calculations within multiple groups were done using ANOVA and Student-Newman-Keuls post hoc test, or ANOVA and Tukey HSD multiple comparison test, as specified in the caption of each figure, with 95% confidence interval using the GraphPad or SigmaPlot software. Significant differences were reported as ***$p < 0.001$ unless reported otherwise in the figures.

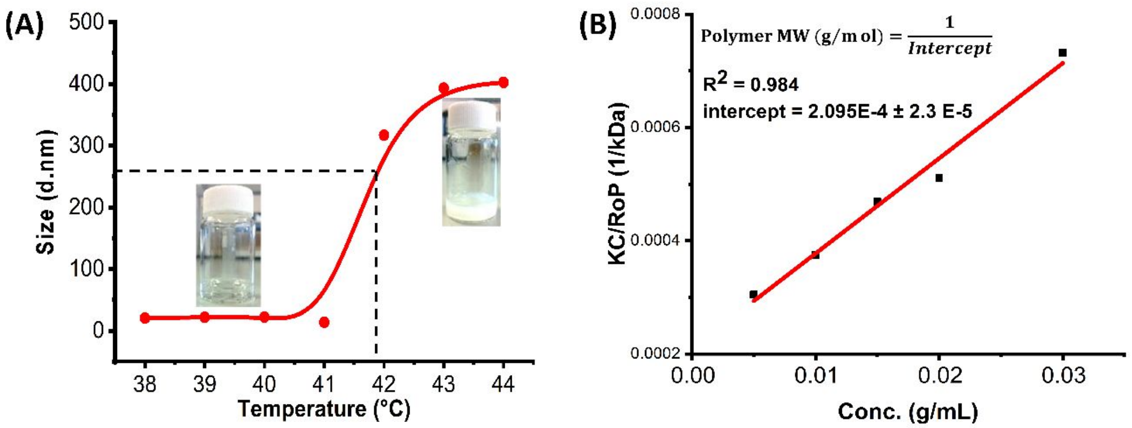

Figure S1. *(A) LCST of TR-polymer measured by DLS measurement of change in hydrodynamic size with temperature and (B) Average TR-polymer molecular weight as determined by Static Light Scattering (SLS) method using Zetasizernano series instrument. The plot shows scattering intensity vs concentration of polymer and by taking the reciprocal of this Debye plot's intercept, the average molecular weight of the polymer was determined.*

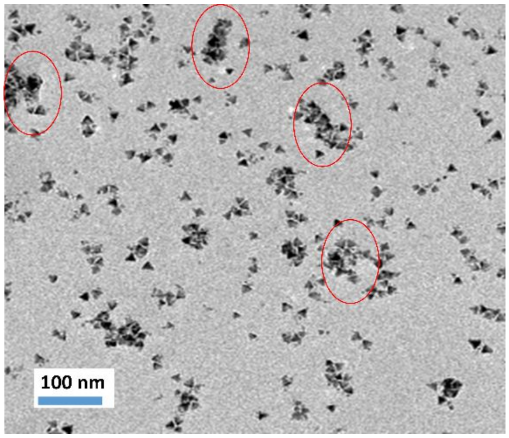

Figure S2. *Typical TEM image showing aggregated TR-$CuFeS_2$ NPs brought in water at a ligand/nm^2 ratio less than 50.*

XPS was employed to compare the changes in surface composition on the pristine $CuFeS_2$ NPs after TR-polymer coating and DOXO loading. The wide scan spectrum (Figure S3A) collected on the pristine $CuFeS_2$ NPs (blue plot) showed the expected signals for the inorganic core (Cu, Fe, and S), together with signals from the organic shell (C, N) and traces of surface oxidation/environmental contamination (O signal). The intensity of the signals related to the inorganic core of the NPs drastically dropped after TR-polymer coating (green line), as shown in the insets of Figure S3A. At the same time, the C and O signals intensity increased. Taking into

consideration the surface sensitivity of XPS and its probing depth (10 nm) this is an indication of the successful TR-functionalization.

The intensity drop of the CuFeS2 related signals is even more relevant after the DOXO loading. As shown in the insets of Figure S3A, Cu 2p peaks almost disappeared, as well as S signals. At the same time, C and O signals further increased in intensity. Again, taking into consideration the surface sensitivity of the technique, the XPS data support the effective DOXO-loading. Effective TR-coating was further supported by the high-resolution XPS data collected on the binding energy regions typical for C 1s and Cu 3p-Br 3d peaks (Figure S3Bi-iii). Indeed, the comparison of the C 1s spectra collected on pristine (panel (i)) and TR-$CuFeS_2$ NCs (panel (ii)) displayed a strong increase in the intensity of C–O (286.3 eV), C=O (287.0 eV) and C(=O)–O (288.7 eV) contributions after the TR-polymer coating. Furthermore, the presence of a Br 3d doublet, with Br $3d_{5/2}$ component at 71.9±0.2 eV (panel (iii)), indicated the presence of TR- on the NPs. High-resolution spectra collected on TR-$CuFeS_2$-DOXO NPs closely resemble those shown in Figure S3B for TR-$CuFeS_2$ and are therefore omitted.

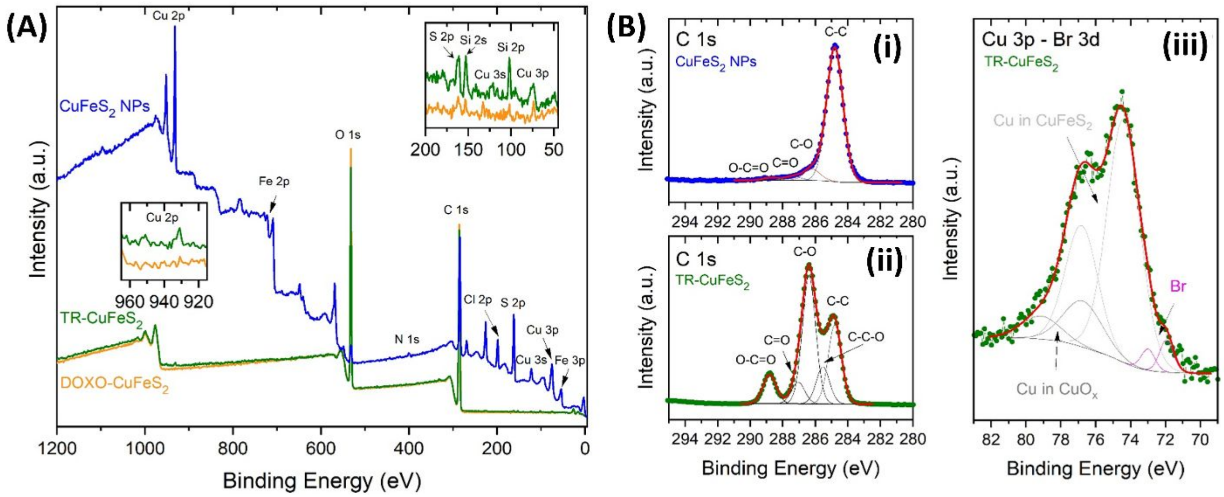

***Figure S3**. Comparative XPS characterization. (A) XPS wide scans collected on pristine $CuFeS_2$ NPs (blue line); polymer coated TR-$CuFeS_2$ NPs (green line) and DOXO-loaded TR-$CuFeS_2$-DOXO NPs (orange line). (B) High resolution XPS spectra, (i) C 1s on pristine CuFeS2 NPs; (ii) C 1s on TR-$CuFeS_2$ NPs, showing increased signal intensities for groups typically found in polymers repeating units; and (iii) data collected on the energy region typical for Cu 3p and Br 3d signals. The presence of a Br doublet supports the presence of TR-polymer (Br element is present as initiator in the polymer structure).*

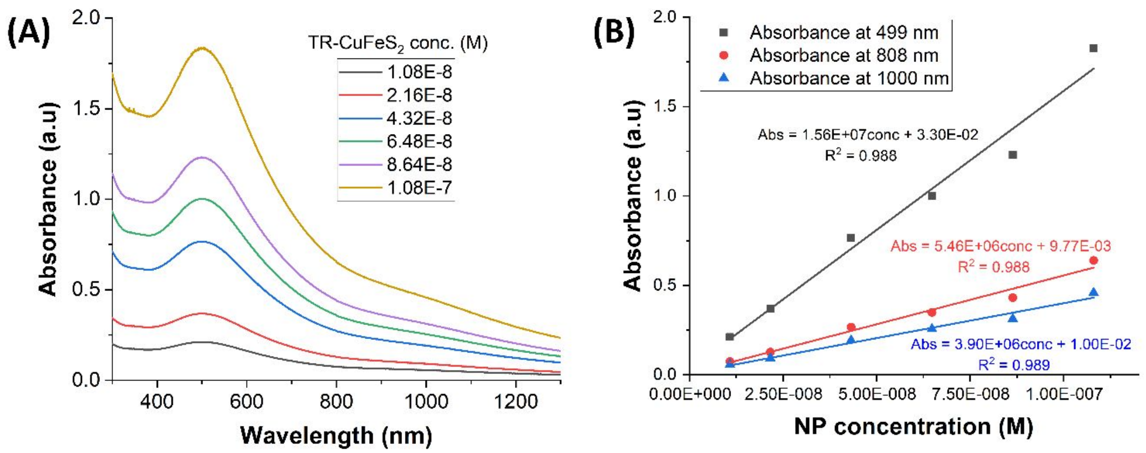

Figure S4. *(A) Extinction spectra of the TR-$CuFeS_2$ NPs at different concentrations acquired at an optical path length of 0.3 cm; (B) Absorbance values at 499, 808 and 1000 nm wavelength were plotted as a function of the TR-$CuFeS_2$ NPs concentrations. The slope of the linear fit of each curve was used to calculate the molar extinction coefficient (ε) at the specified wavelength.*

Table S1. *Molar extinction coefficient of TR-$CuFeS_2$ NPs at different wavelengths.*

	499 nm	808 nm	1000 nm
slope	1.56E+07	5.46E+06	3.90E+06
epsilon M^{-1} cm^{-1}	5.20E+07	1.82E+07	1.30E+07

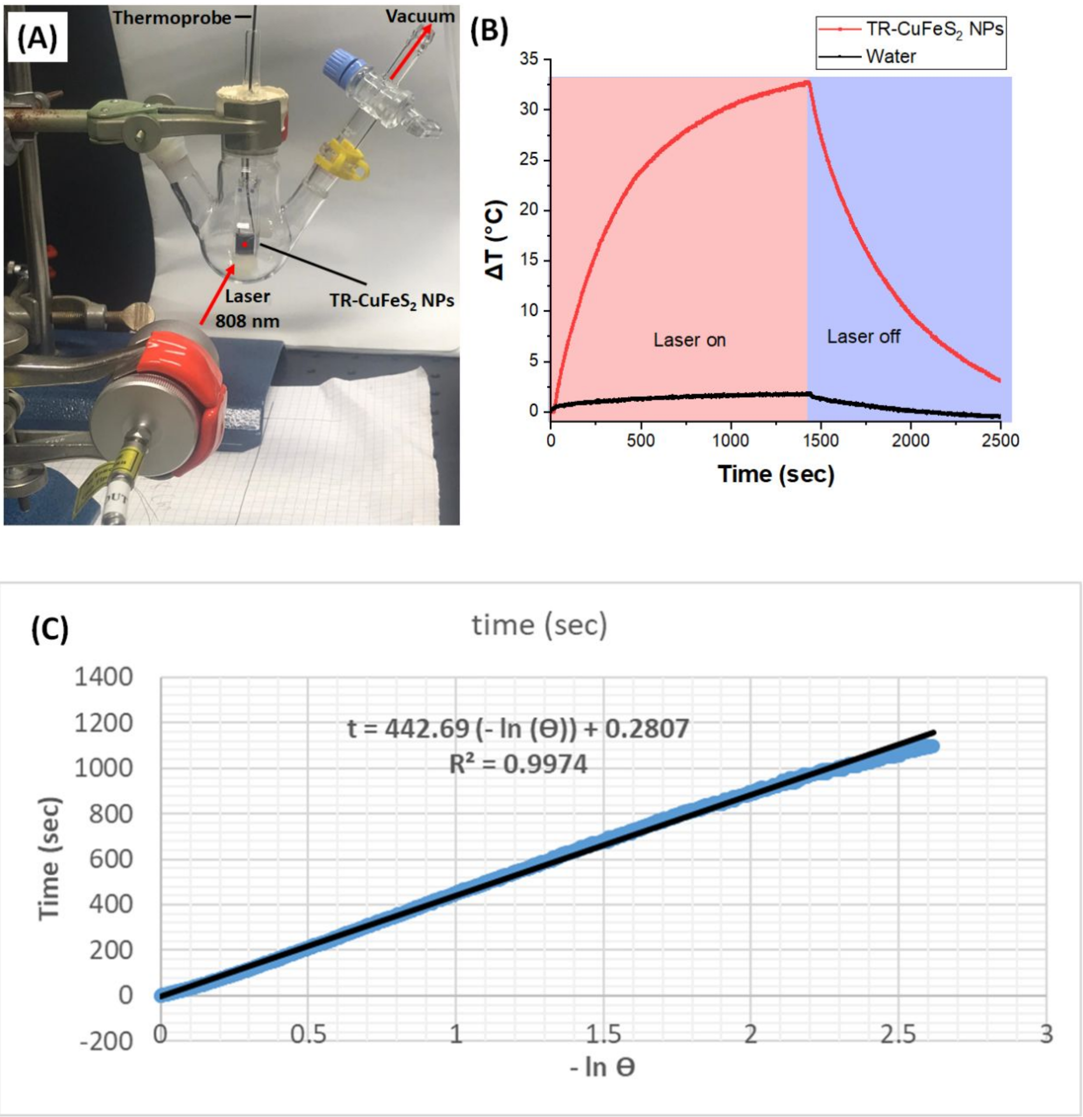

Figure S5. *(A) The set-up used for the photothermal conversion efficiency determination. A self-made irradiation chamber was designed using a three neck round bottom flask into which a quartz cuvette connected with a glass tube was inserted. Fitted in this chamber also is a thermoprobe to measure the temperature of the NPs solution during laser 808 nm irradiation and (B) Heating-cooling cycle of TR-*$CuFeS_2$ *NPs solution (red curve) and Milli-Q water (black curve) and (C) Linear fit time/-ln(θ) of the cooling step of TR-*$CuFeS_2$ *NP solution.*

Table S2*: Absorbance at irradiation wavelength (*Abs_{808nm}*), mass of NP solution (m sol), increasing temperature after laser irradiation (ΔT), time system constant (*τ_s*), thermal conductance (hS), lase power (I) and photothermal conversion efficiency (Conversion. eff).*

A808 nm	m sol (g)	ΔT (°K)	τ_s (s)	*h* S (W K^{-1})	Laser power I (W)	Conversion eff. (%)
0.574	0.9661	32.8	442.69	0.02	0.753	47.8

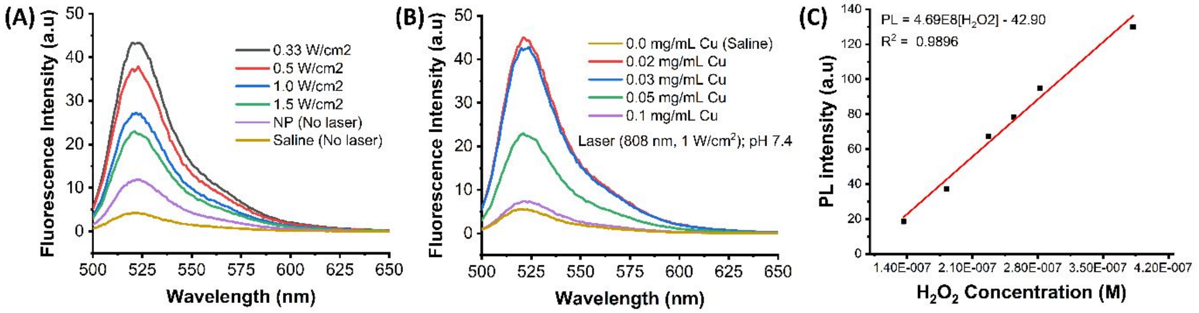

Figure S6. *Photoluminescence spectra (Excitation 485 and emission 523 nm) related to the amount of ROS generated by TR-CuFeS$_2$ NPs upon laser 808 nm irradiation at different laser power densities (A) and NP dose s(B). (C) Hydrogen peroxide dose versus PL intensity at 523 nm calibration plot used to quantify the amount of ROS generation using 5 µM DCFH dye/HRP solution.*

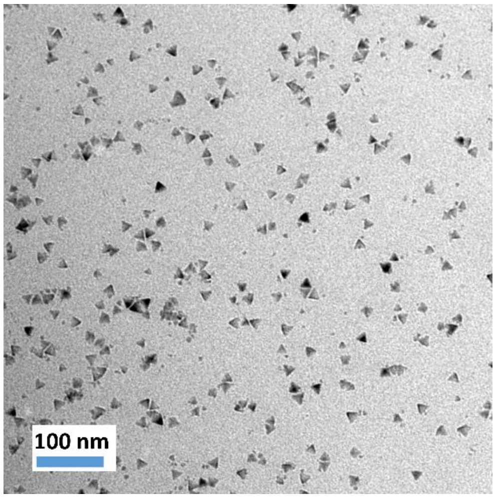

Figure S7. *TEM image showing aged TR-CuFeS$_2$ NPs after leakage of 7.6% iron (Fe) from its core – their pyramidal shape does not look significantly affected.*

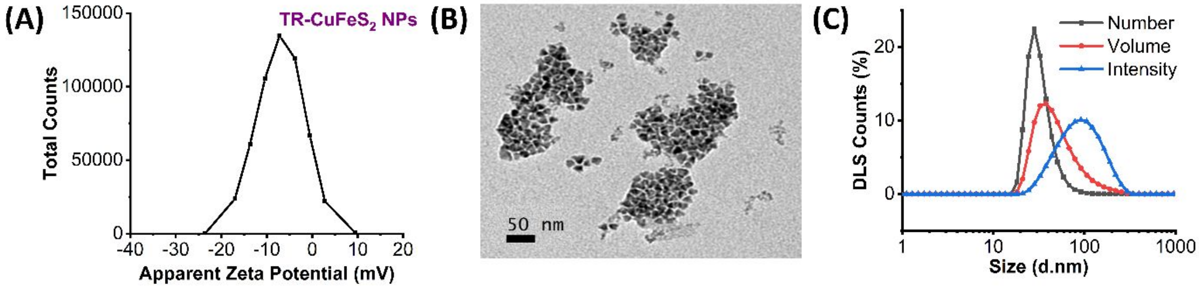

Figure S8. *(A) Representative zeta potential plot of TR-CuFeS$_2$ NPs before DOXO loading; (B) TEM image showing aggregated TR-CuFeS$_2$ Dox NPs obtained when Doxorubicin loading was conducted for a 14 h reaction time and (C) DLS traces of hydrodynamic size weighted by intensity, volume and number for TR-CuFeS$_2$ DOXO NPs obtained at the optimized loading condition (6 h loading time, Milli-Q water pH 7.4).*

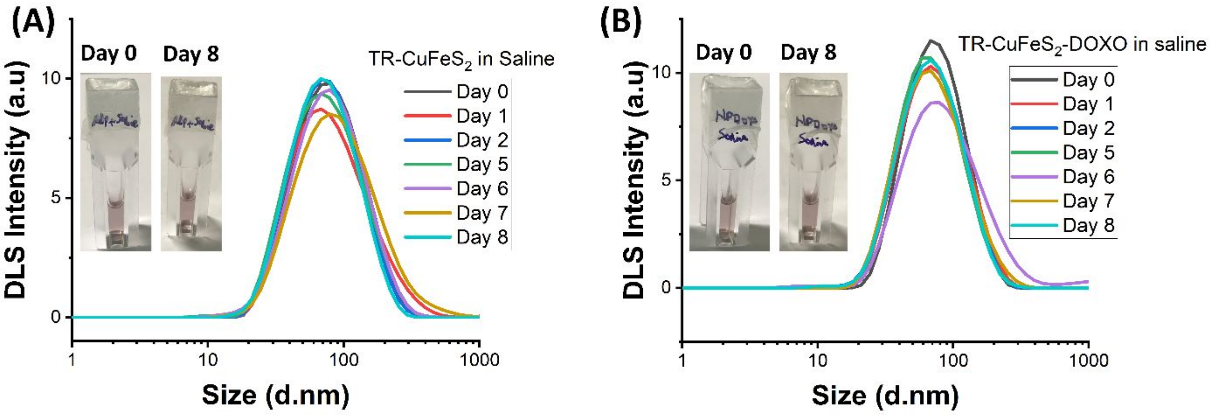

Figure S9. *Hydrodynamic Stability of TR-CuFeS$_2$ and TR-CuFeS$_2$-DOXO NPs in physiological conditions. The DLS by intensity traces of 0.04 mg/mL [Cu] TR-CuFeS$_2$ (A) and 0.04 mg/mL [Cu] TR-CuFeS$_2$-DOXO NPs (B) dispersed in 0.9% Saline solution at day 0 up to day 8, storage at ambient condition. The insets show images of the NP solutions in cuvettes as observed under visible light at day 0 and 8 respectively.*

Table S3*. DOXO Loading Quantification*

Sample No.	[Cu] NPs Mg/mL	[Doxo]$_{ini}$ Mg/mL	% Doxo Encapsulated*	Wt % Loading**
1	0.05	0.02	29.09	10.39
2	0.05	0.02	27.15	9.70
3	0.1	0.05	27.55	7.87
4	0.18	0.1	30.25	9.60
5	0.21	0.1	31.18	8.66
	**Average**		**29.17**	**9.24**
	**Std. Dev.**		**±1.93**	**±0.98**

$* \ \% \ Doxo \ encapsulated = \frac{[\mathrm{Dox}]\mathrm{encap}}{[\mathrm{Dox}]\mathrm{ini}} * 100; \ ** \ \mathrm{Wt} \ \% = \frac{[\mathrm{Dox}]\mathrm{encap}}{[\mathrm{Cu}] \ \mathrm{NPs}} * 100$

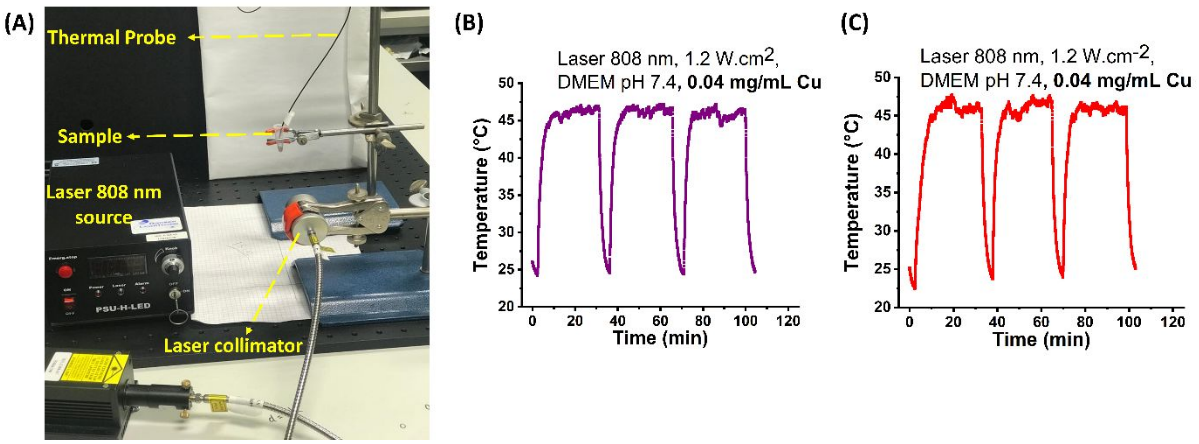

***Figure S10**. (A) The photo of laser 808 nm setup used in our study of in vitro Photothermal, ROS and cellular experiments (B) heating profile of treatment of U87 cells with pristine TR-CuFeS_2 (purple plot) and (C) heating profile of treatment of U87 cells with TR-CuFeS_2 Doxo NPs (red plot).*

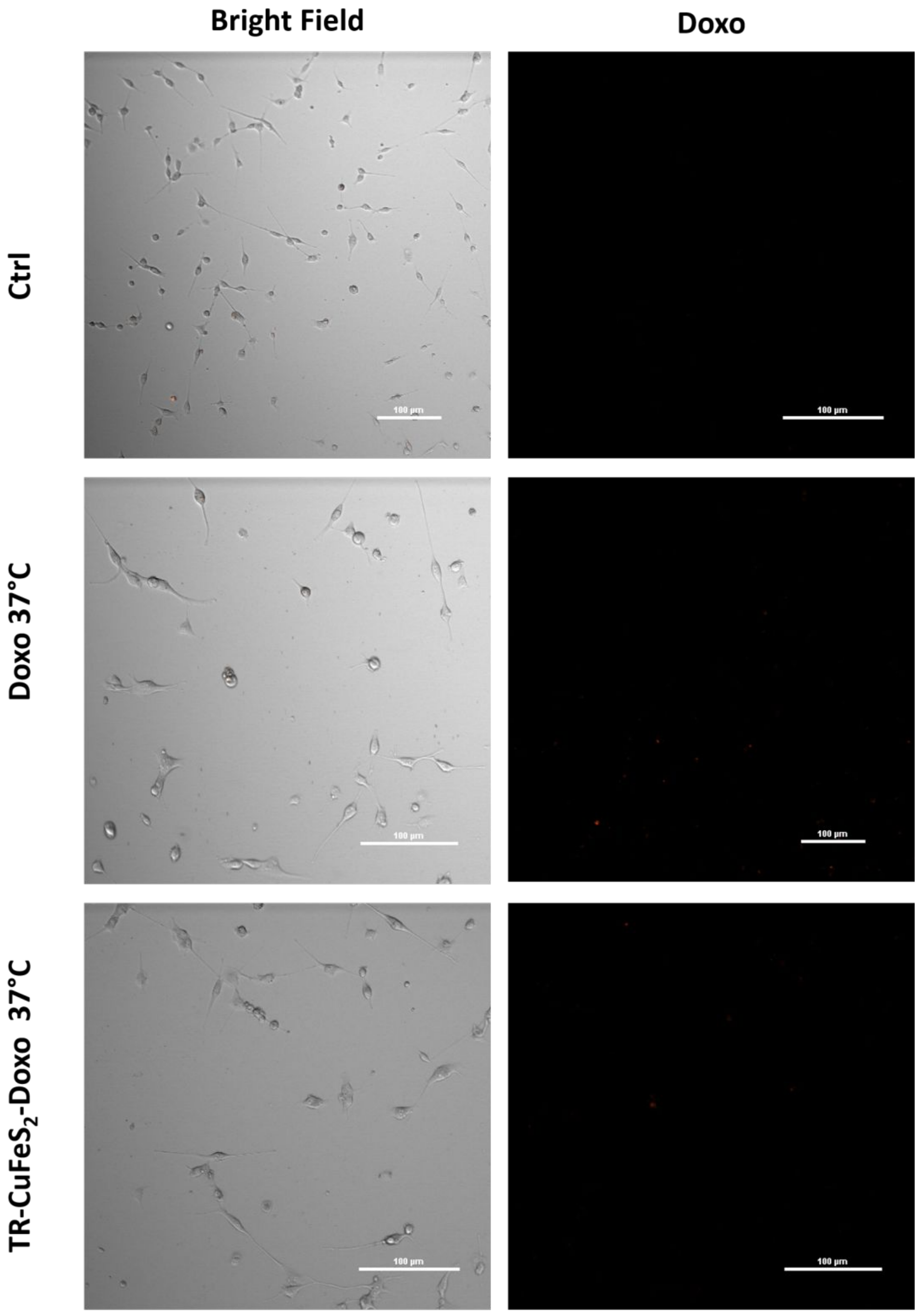

Figure S11. *Confocal images of just U87 cells used as a control (first row), U87 cells incubated with free DOXO at 37°C (second row) and U87 cells incubated with TR-$CuFeS_2$ DOXO NPs without laser application (third row). In the column on the left are shown the bright field images, while that on the right column the red channel that should show red dots in case of DOXO internalization within the cells. Scale bar: 100µm.*

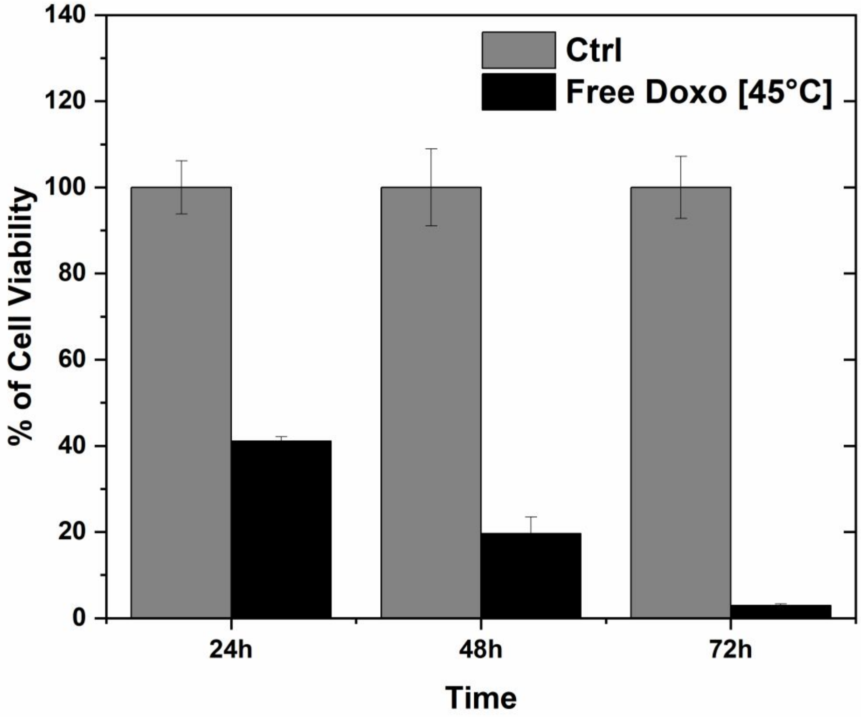

Figure S12. *Cell Viability of U87 after incubation with free DOXO at the released concentration and exposed in a water bath at 45°C for one hour and a half (in black). A sample of U87 cells was used as a control (in grey). The viability was evaluated through Trypan Blue exclusion method at 24 h, 48 h and 72 h after the treatment.*

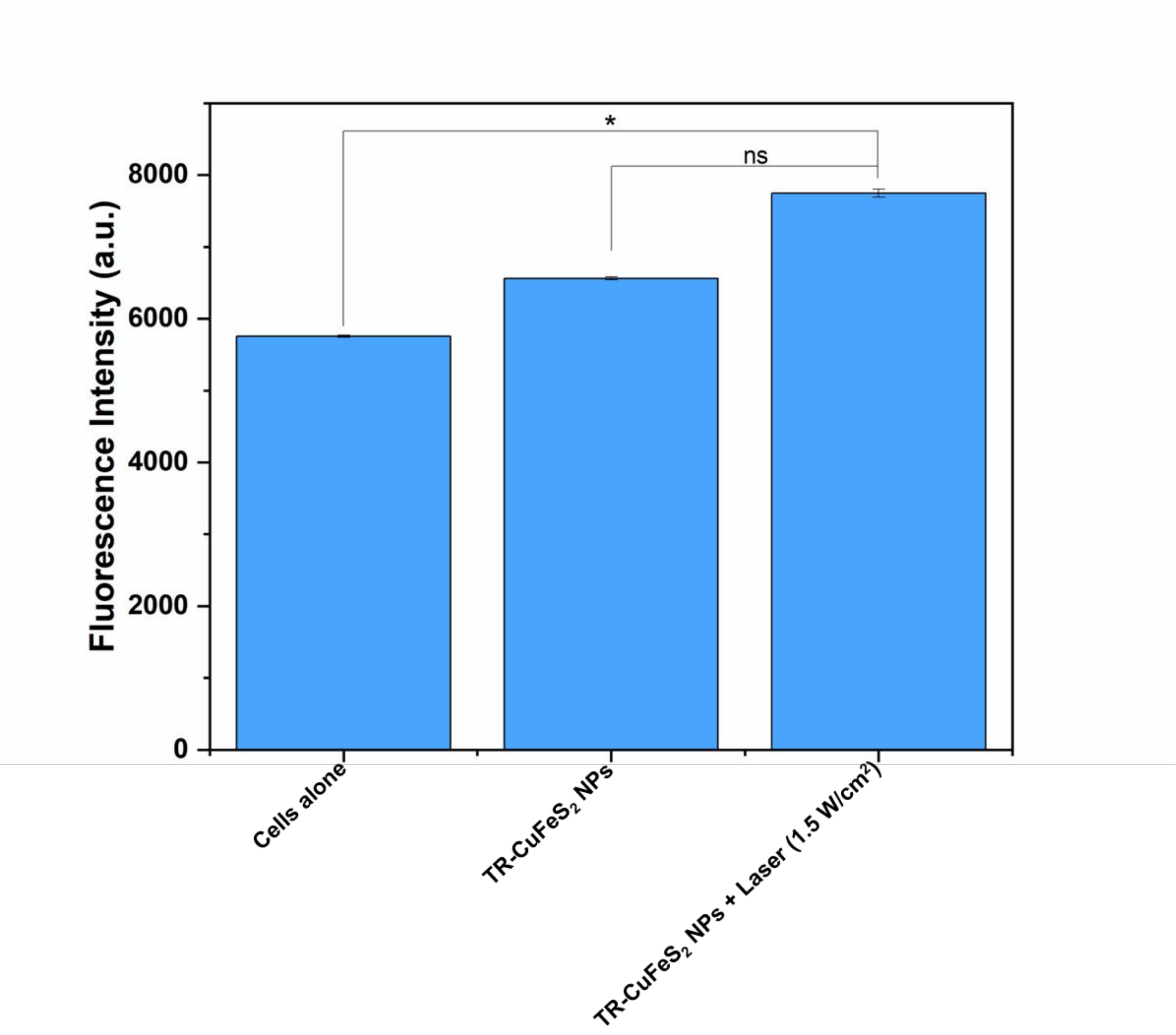

Figure S13. *Proofs of the ROS production in A431 cancer cells. Cells were either incubated with TR-CuFeS_2 NPs without exposure to laser or with exposure to laser at 1.5 W/cm^2 for 5 minutes Next, the sample were incubated with the dye DCFHDA for the evaluation of produced ROS by the intensity of the DCF dye production. The activity of ROS generated proportional to ROS production, was measured by the fluorescent intensity of DCF evaluated through a fluorescent plate reader. Values are presented as mean with error bars indicating the standard deviation (SD) for n = 3 independent experiments. Statistical analysis was performed using a one−way ANOVA test (* 0.01 < p < 0.05).*

References

(1) Ghosh, S.; Avellini, T.; Petrelli, A.; Kriegel, I.; Gaspari, R.; Almeida, G.; Bertoni, G.; Cavalli, A.; Scotognella, F.; Pellegrino, T.; Manna, L. Colloidal CuFeS2 Nanocrystals: Intermediate Fe d-Band Leads to High Photothermal Conversion Efficiency. *Chem. Mater.* **2016**, *28* (13), 4848–4858.

(2) Roper, D. K.; Ahn, W.; Hoepfner, M. Microscale Heat Transfer Transduced by Surface Plasmon Resonant Gold Nanoparticles. **2007**. https://doi.org/10.1021/jp064341w.

(3) Zhao, J.; Riediker, M. Detecting the Oxidative Reactivity of Nanoparticles: A New Protocol for Reducing Artifacts. *J. Nanoparticle Res.* **2014**, *16* (7).

(4) Hopke, P. K.; Wang, Y.; Sun, L.; Chalupa, D. C.; Utell, M. J. Laboratory and Field Testing of an Automated Atmospheric Particle-Bound Reactive Oxygen Species Sampling-Analysis System. *J. Toxicol.* **2011**, *2011*.
